# HTPI: A New Head–Tail Population Initialization for Feature Selection Stability in IoT IDSs with Post Hoc Explainable AI Analysis

**DOI:** 10.3390/s26165207

**Published:** 2026-08-17

**Authors:** Saud Abdullah Alzughaibi, Iftikhar Ahmad, Madini Alassafi

**Affiliations:** Department of Information Technology, Faculty of Computing and Information Technology, King Abdulaziz University, Jeddah 21589, Saudi Arabia; iakhan@kau.edu.sa (I.A.); malasafi@kau.edu.sa (M.A.)

**Keywords:** internet of things, intrusion detection system, feature selection, head–tail population initialization, AHGA-SA, genetic algorithm, simulated annealing, metaheuristic optimization, post hoc explainable artificial intelligence

## Abstract

Stochastic metaheuristic feature selection (FS) may generate unstable feature subsets across repeated runs, undermining reproducibility in Internet of Things (IoT) intrusion detection systems (IDSs). This paper presents Head–Tail Population Initialization (HTPI), a feature-importance-guided initialization approach for enhancing the stability of Adaptive Hybrid Genetic Algorithm–Simulated Annealing (AHGA-SA)-based FS. HTPI uses feature-importance scores to split candidate features into Head and Tail groups and initializes candidate subsets by prioritizing Head features and sampling Tail features with importance-based weights. HTPI is integrated into AHGA-SA as an incremental extension, termed HTPI-AHGA-SA, and modifies only the initialization and reinitialization steps. Experiments on eight IoT-oriented IDS datasets using 50 runs per configuration, with seeds paired across methods, showed significantly higher Nogueira stability under HTPI-AHGA-SA on all datasets after Holm correction, with non-overlapping 95% leave-one-run-out jackknife confidence intervals in every case. These results characterize algorithmic cross-run stability under a fixed data partition. All absolute differences in dataset-level mean F1 Macro remained below 0.003; formal equivalence at this margin was supported for six datasets, while dataset-specific security-metric trade-offs remained. On three representative datasets, post hoc explainable artificial intelligence (XAI) analyses indicated generally higher permutation importance (PI)-based cross-run consistency and measurable predictive utility in the selected Head and Tail portions under retraining.

## 1. Introduction

The rapid growth of Internet of Things (IoT) systems has increased the exposure of connected devices, sensors, gateways, and cyber-physical infrastructures to diverse network attacks. IoT-oriented environments, such as the Internet of Medical Things (IoMT), Industrial Internet of Things (IIoT), smart-home systems, Agricultural IoT (Ag-IoT), and Internet of Vehicles (IoV), typically generate heterogeneous and high-dimensional traffic data. Machine learning (ML)-based intrusion detection systems (IDSs) can learn discriminative patterns from such data, but redundant, irrelevant, or weakly informative features can lead to higher computational costs, more complex model training, and reduced interpretability of the resulting detection model [1,2,3].

Feature selection (FS) is therefore an important preprocessing step for IDS development, as it can reduce dimensionality while maintaining or improving detection performance by selecting compact and informative feature subsets [4,5]. However, FS is a combinatorial optimization problem because the number of possible feature subsets increases exponentially with the number of candidate features [6]. For this reason, metaheuristic algorithms (MAs) have been extensively used in wrapper and hybrid FS, as they can search large feature spaces and evaluate candidate subsets using classifier feedback [7].

Recent IoT-oriented IDS studies increasingly combine FS with deep learning and explainable artificial intelligence (XAI) to improve detection performance, computational efficiency, and interpretability. Transformer- and large language model-based approaches have also been explored for traffic representation, feature reduction, and data augmentation. However, these studies mainly evaluate predictive performance, efficiency, and interpretability rather than the reproducibility of selected feature subsets across stochastic runs [2,8,9].

FS stability is a separate reliability concern because different samples, algorithms, or stochastic runs may produce different feature subsets with similar predictive performance [10,11]. This concern is particularly relevant to metaheuristic FS because of its stochastic search process [12]. In IDS applications, selected features should also be reproducible and interpretable across repeated runs [13,14]. Stability should therefore be evaluated alongside predictive performance and subset size [15,16].

In our previous work, we proposed the original adaptive hybrid genetic algorithm–simulated annealing (AHGA-SA) framework as an adaptive hybrid FS method for IoT-oriented intrusion detection [17]. AHGA-SA combines Extreme Gradient Boosting (XGBoost)-based preselection [18], genetic algorithm (GA)-based global exploration [19], and simulated annealing (SA)-based local refinement [20]. The candidate feature space is reduced using XGBoost importance scores before the AHGA-SA search starts, but the initial population is still generated randomly. As a result, the ranking information obtained during preselection is not directly used when constructing the initial candidate population, which may contribute to run-to-run variability in the selected subsets.

Population initialization is a design component that can influence early search behavior, diversity, convergence, and final solution quality. Accordingly, several initialization strategies have been proposed, including random, quasi-random, chaotic, opposition-based, probability-distribution-based, clustering-based, hybrid, elite-based, and adaptive designs [21,22,23]. Most of these strategies, however, are designed for general optimization settings rather than for feature-importance-guided initialization within the fixed two-stage search design of the AHGA-SA framework. This motivates a focused investigation of how the ranked feature pool already produced by AHGA-SA can be used more effectively during population construction.

To address this issue, this study proposes Head–Tail Population Initialization (HTPI), a feature-importance-guided initialization strategy for AHGA-SA-based FS. HTPI divides the preselected feature pool into a Head group containing the highest-ranked features and a Tail group containing the remaining candidate features. Population construction prioritizes Head features and samples Tail features without replacement using importance-based probabilities, thereby directing the search toward higher-ranked regions while retaining non-zero sampling opportunities for lower-ranked Tail features.

This study presents HTPI as an incremental extension of AHGA-SA rather than as a standalone FS framework. It modifies only the Stage-1 population initialization and Stage-2 reinitialization, while all other AHGA-SA components remain unchanged. The resulting configuration is termed HTPI-AHGA-SA. The conclusions are therefore limited to this evaluated configuration. Its specific contribution is the use of the existing feature ranking to guide Head–Tail population construction and improve cross-run FS stability while retaining importance-weighted sampling opportunities from the Tail set.

The main contributions of this study are as follows:

- A new feature-importance-guided Head–Tail population construction strategy is introduced, in which Head features are prioritized, and Tail features are sampled without replacement according to their importance.
- A repeated-run evaluation is conducted on eight IoT-oriented IDS datasets using 50 runs per configuration with paired seeds 1–50 and fixed data splits, comparing Original AHGA-SA and HTPI-AHGA-SA in terms of selected-feature count, runtime, predictive performance, and FS stability.
- A formal paired evaluation is conducted using Nogueira’s size-corrected stability, leave-one-run-out jackknife confidence intervals (CIs), paired pseudo-value tests, Cohen’s $d_{z}$, and Holm correction. Predictive differences are assessed using paired Wilcoxon tests and F1 Macro equivalence, while post hoc XAI examines cross-run consistency based on permutation importance (PI) and retraining-based group sufficiency.

The remainder of this paper is organized as follows. Section 2 reviews related work on metaheuristic FS, AHGA-SA, and population initialization. Section 3 describes the HTPI integration and experimental protocol. Section 4 reports the stability, predictive performance, sensitivity, and targeted component-ablation results. Section 5 presents the post hoc XAI analyses. Section 6 discusses the findings and limitations. Finally, Section 7 concludes the paper and outlines future directions.

## 2. Related Work

This section situates HTPI within the relevant research landscape and provides the context for its methodological contribution.

### 2.1. Metaheuristic FS for Intrusion Detection

Several studies have employed hybrid metaheuristic FS techniques to improve intrusion-detection performance. Kareem et al. proposed a hybrid Gorilla Troops Optimizer and Bird Swarm Algorithm FS model for IoT intrusion detection [24]. In a related direction, Zheng et al. introduced a hybrid whale-optimization-based FS method that combines complementary search mechanisms for FS [25]. Similarly, Akinola et al. proposed a hybrid binary metaheuristic approach with SA for high-dimensional FS [26]. These works show that combining complementary search mechanisms can improve exploration, exploitation, and predictive performance in FS tasks.

The IDS-oriented studies reviewed above emphasize classification performance and subset reduction, while cross-run feature-selection stability receives less attention. This matters because two FS methods can achieve similar predictive performance while producing substantially different feature subsets, affecting reproducibility, interpretability, and deployment trust. FS stability should therefore be evaluated alongside predictive performance and compactness in IDS applications.

### 2.2. Feature-Ranking-Guided and AHGA-SA-Based FS

Hybrid filter–wrapper FS methods often use feature-ranking information to guide the metaheuristic search because filter scores are computationally inexpensive, while the wrapper stage can still capture feature interactions. In high-dimensional microarray data, Apolloni et al. constructed part of the initial population from top-ranked features and generated the remainder randomly to preserve diversity [27]. Deniz and Kiziloz showed that information-gain ranking yields better initial populations than random generation, particularly for medium- and large-sized datasets [28]. Xue et al. used filter-derived heuristics with mixed initialization for particle swarm optimization (PSO) [29], while Wang et al. used rankings based on principal component analysis (PCA) to initialize PSO [30]. Collectively, these studies show that filter scores can guide population construction while randomized individuals maintain broader search-space coverage.

A recent large-scale FS study also highlighted the importance of initialization. Xu et al. proposed a hybrid initialization and effective reproduction scheme for bi-objective large-scale FS, using hybrid initialization to improve the initial population and effective reproduction to generate offspring during the search [31]. This work supports the use of feature-ranking information not only to reduce the feature space but also to construct a higher-quality initial population.

The ranking-guided studies reviewed above mainly use feature rankings to seed an initial population or combine ranked and random individuals. HTPI instead reuses the XGBoost ranking already produced within AHGA-SA and applies Head prioritization and importance-weighted Tail sampling to both Stage-1 initialization and Stage-2 reinitialization. Unlike elite-based initialization, HTPI does not copy previously optimized elite masks into the population; Stage 2 reuses only their feature counts to construct new Head-prioritized masks. It is therefore a problem-specific extension of AHGA-SA rather than a general replacement for other initialization methods.

The original AHGA-SA framework combines XGBoost-based preselection, GA-based global exploration, SA-based local refinement, duplicate removal, and a fixed two-stage search, while retaining random population initialization within the preselected feature space [17].

### 2.3. Population Initialization in Metaheuristic Algorithms

The initialization of the population is an important design aspect in population-based MAs, as it can affect the initial search behavior, population diversity, convergence rate, and solution quality [21,22]. In this context, Li et al. systematically compared 22 initialization methods across differential evolution, PSO, cuckoo search, artificial bee colony, and GA, and found that the effect of initialization is algorithm- and problem-dependent, and that some probability distributions can enhance performance under certain conditions [32]. Initialization is therefore not a neutral implementation detail.

Initialization research also includes quasi-random, distribution-based, and clustering-based strategies. Space-filling and low-discrepancy designs, including Latin hypercube sampling and Halton and Sobol sequences, have been investigated to improve search-space coverage relative to uniform random initialization [33,34]. Alternative probability distributions can bias initial populations in different ways [32,35], while clustering- and Cauchy-based schemes can concentrate candidates in promising regions while retaining exploratory variation [36].

Because HTPI modifies initialization within the GA-based AHGA-SA framework, GA studies outside IDS FS also provide relevant motivation for problem-specific initialization. In scheduling, Vlašić et al. applied dispatching-rule-based initialization to enhance GA performance [37]. Hassanat et al. introduced a regression-based initialization mechanism for GA in the context of the traveling salesman problem [38]. For vehicle routing, Li et al. improved the population initialization of a reinsertion GA for the electric vehicle routing problem with time windows, using a neighborhood-search-based strategy to obtain high-quality initial routes [39]. These studies collectively reinforce the general notion that problem-related information can be helpful in the initialization phase, especially when random initialization yields infeasible, low-quality, or poorly distributed initial solutions.

The initialization studies reviewed above primarily address general benchmark functions, continuous optimization, scheduling, or general population-based metaheuristics. FS for IDSs has a specific binary subset structure, and population construction must also account for how features are distributed within candidate subsets. An initialization strategy for IDS FS can therefore use feature-importance information while retaining opportunities to explore alternative feature combinations.

### 2.4. Motivation for the Proposed HTPI

The reviewed literature reveals a specific methodological gap: ranking-guided initialization studies do not explicitly target cross-run FS stability within the fixed two-stage AHGA-SA search design, while Original AHGA-SA produces a ranked candidate feature pool but retains random population construction. HTPI addresses this gap by reusing the existing XGBoost ranking to guide Head–Tail population construction during both Stage-1 initialization and Stage-2 reinitialization, without altering the remaining AHGA-SA search components.

## 3. Methodology

**Figure 1:**
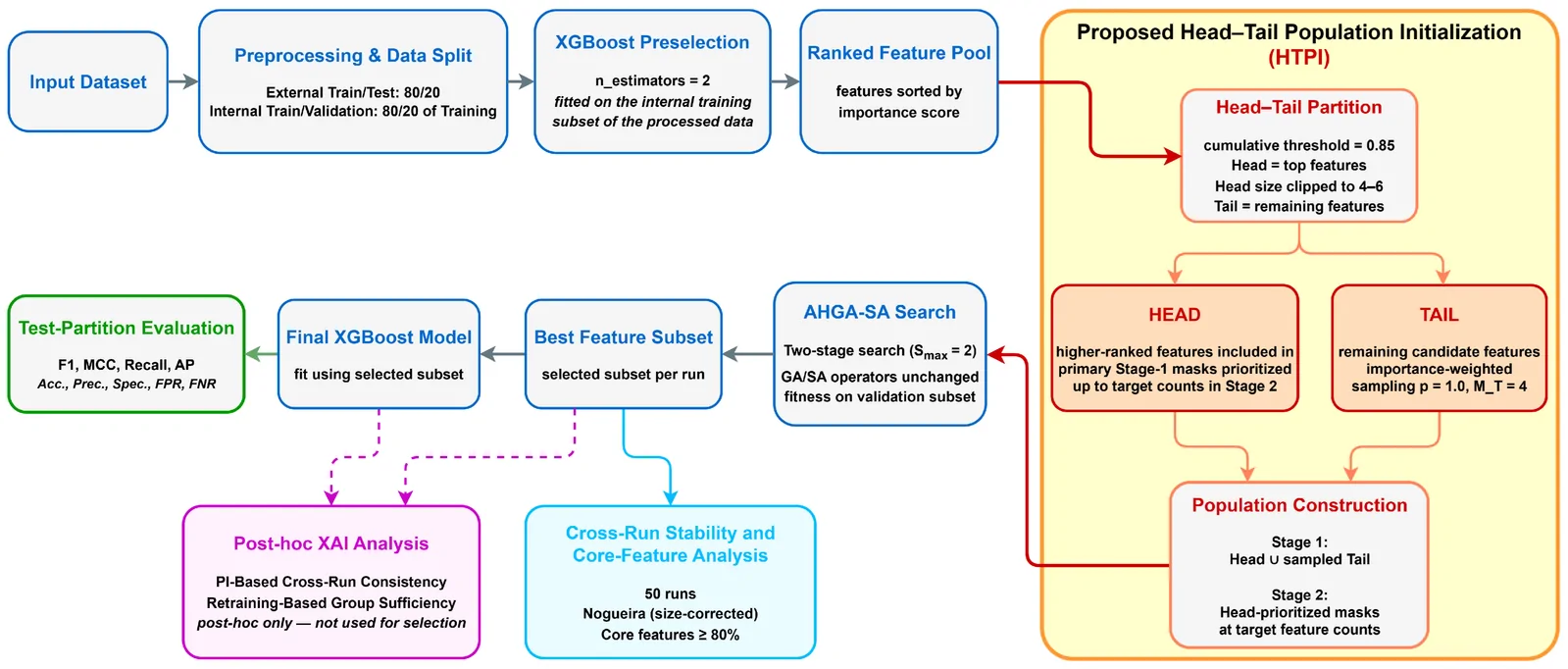
Overview of the HTPI-AHGA-SA pipeline. AHGA-SA: Adaptive Hybrid Genetic Algorithm–Simulated Annealing; HTPI: Head–Tail Population Initialization; XGBoost: Extreme Gradient Boosting; GA: genetic algorithm; SA: simulated annealing; XAI: explainable artificial intelligence; PI: permutation importance; MCC: Matthews correlation coefficient; AP: attack-class average precision; Acc.: accuracy; Prec.: macro-averaged precision; Spec.: specificity; FPR: false-positive rate; FNR: false-negative rate. In the evaluation box, F1 and Recall denote macro-averaged metrics; $S_{\max}$ is the maximum number of search stages, *p* is the Tail-weight power, and $M_{T}$ is the Stage-1 maximum Tail budget.

This section outlines the data preprocessing, XGBoost-based preselection, integration of HTPI into AHGA-SA, and the evaluation protocol for comparing Original AHGA-SA and HTPI-AHGA-SA. This pipeline is summarized in Figure 1.

### 3.1. Data Preprocessing and Benchmark Datasets

HTPI-AHGA-SA was evaluated on eight public intrusion-detection datasets from diverse IoT domains. IoTID20 [40,41] represents general IoT botnet traffic. WUSTL-EHMS [42,43] and IoMT-TrafficData [44,45] represent IoMT environments. The ML version of Edge-IIoTset, denoted Edge-IIoTset (ML) [46,47], and X-IIoTID [48,49] cover IIoT scenarios. RT-IoT2022 [50,51] contains real-time smart-home IoT traffic, Farm-Flow [52,53] represents an Ag-IoT environment, and CICIoV2024 [54,55] represents IoV network traffic.

These datasets enable the evaluation of HTPI-AHGA-SA under diverse traffic characteristics, feature dimensionalities, and class distributions. Sample counts, initial and processed feature counts, preselected candidate-pool sizes, and class-distribution statistics are given in Table 1.

To ensure direct comparability with the original AHGA-SA study [17], the preprocessing pipeline for IoTID20, WUSTL-EHMS, and Edge-IIoTset (ML) retained the feature-removal and transformation procedures used in that study. For the other five datasets, namely RT-IoT2022, IoMT-TrafficData, Farm-Flow, X-IIoTID, and CICIoV2024, a uniform pipeline for removing identifier, alternate-label, and verified leakage columns was used because no existing AHGA-SA baseline was available for these datasets. Structural or session-identifier columns and detector-output or label-leakage columns were removed by name or verified leakage behavior, where applicable. Target and alternate-label columns were excluded before feature counting and are therefore not included in the removed-feature counts. The complete list of removed feature columns for all eight datasets is given in Table A1 in Appendix A.

For CICIoV2024, the removed ID- and DATA-prefixed columns listed in Table A1 are constant-valued Controller Area Network (CAN) bit-level columns. They were removed by the constant-column rule applied to the CICIoV2024 training set. They are distinct from the retained non-constant processed CAN bit-level features with similar prefixes, such as textttID8, ID10, ID11, ID13, DATA_310, and DATA_611.

Positive and negative infinite values were replaced with missing values. In X-IIoTID, 40 otherwise numerical feature columns contained dash symbols (-) used as missing-value placeholders and were therefore read as object-valued columns. These columns were converted to numeric form before data splitting, with those dash entries coerced to missing values. After splitting, the resulting missing entries were imputed using medians derived from the external training partition.

For IoTID20, WUSTL-EHMS, and Edge-IIoTset (ML), the original AHGA-SA preprocessing implementation was retained for direct comparability: numerical median imputation and label encoding of object-valued feature columns were applied before the fixed external train/test split. Separate numerical standardizers were then fitted on the external training and internal training subsets and applied to the corresponding test and validation partitions. For the remaining five datasets, numerical medians were derived from the external training partition, and constant-valued and exact-duplicate feature columns were identified using that partition. Separate numerical standardizers were fitted on the external training and internal training subsets and applied to their corresponding test and validation partitions.

Among the five added datasets, only RT-IoT2022 (proto, service) and X-IIoTID (Protocol, Service) retained categorical feature columns after the removal of target, alternate-label, identifier, and leakage columns. Missing categorical entries were assigned an explicit missing category before splitting. Separate integer-encoding maps were fitted on the external training and internal training subsets and applied to the corresponding test and validation partitions. A category absent from the fitting partition would have been assigned a single reserved index; however, no unseen category occurred in the evaluated splits. All experiments were conducted under a binary classification setting. Seven datasets already provided binary target labels; RT-IoT2022 was converted to binary classification by treating Thing_Speak, MQTT_Publish, and Wipro_bulb as normal traffic and all remaining attack-type labels as attacks.

Within each dataset, Original AHGA-SA and HTPI-AHGA-SA used identical processed feature matrices, fitted preprocessing transformations, and data partitions.

### 3.2. Original AHGA-SA

AHGA-SA is an adaptive hybrid FS framework that was previously proposed for IoT-oriented intrusion detection. It combines XGBoost-based feature preselection, GA-based global exploration, SA-based local refinement, duplicate removal, and a fixed two-stage search structure with three fitness-evaluation points in each stage. Candidate solutions are encoded as binary feature masks and optimized using an internal training/validation split. Within the original AHGA-SA protocol, the external test partition is excluded from fitness evaluation and subset selection and is used only for final predictive evaluation after the dataset-specific preprocessing described above.

The original framework first ranks features with a lightweight XGBoost model and retains only those with non-zero importance, thereby reducing the search space. The GA component explores alternative subsets through selection, crossover, and mutation, while the SA component refines candidates through local neighborhood search and probabilistic acceptance. The search proceeds over two fixed stages: Stage 1 searches the reduced feature space, and Stage 2 re-searches the subset sizes found promising in Stage 1. Duplicate individuals are removed to avoid redundant evaluations and retain distinct candidate masks. The full algorithmic description and evaluation are given in our previous work [17]; in this study, Original AHGA-SA serves as the baseline.

### 3.3. Proposed HTPI

HTPI replaces only AHGA-SA’s population initialization and reinitialization steps, leaving all other components unchanged. HTPI uses the feature-importance vector produced by the two-estimator XGBoost preselection step. The *d* preselected candidate features are sorted in descending order of importance and divided into a Head set H and a Tail set T by the HeadTailPartition procedure. The Head size is the smallest number of top-ranked features whose cumulative importance reaches the threshold θ (adopted value: 0.85). It is then clipped to the range $\left[H_{\min},H_{\max}\right]$ to prevent very small or very large Head groups. Features not included in the Head group are assigned to the Tail set. The partition was derived once for each dataset from its fixed preselection ranking and reused across the 50 runs. The top-10 XGBoost preselection rankings and the corresponding Head–Tail group assignments are presented in Table A2 in Appendix B.

The probabilities of sampling Tail features are importance-weighted, and sampling is performed without replacement. The sampling probability for each Tail feature *i* is proportional to $w_{i}^{\;p}$, where $w_{i}$ is the importance score of the feature and *p* is the tail-weight power (adopted value: 1.0). A small floor ε prevents very low-importance features from receiving zero or near-zero sampling probability. This design assigns higher sampling probabilities to more important Tail features while retaining non-zero sampling probability for lower-ranked candidates.

HTPI operates within AHGA-SA’s two-stage search structure. In Stage 1, the main HTPI-generated population masks include the Head set and are supplemented by sampling various Tail subsets up to the effective Tail budget. Two seed masks are constructed: Head-only and Head-plus-maximum-Tail-budget. The remaining masks are generated by drawing a Tail cardinality up to the effective Tail budget $M_{T}^{\prime }$ and sampling the corresponding Tail features using WeightedTailSample. If the procedure cannot generate enough distinct masks, the original AHGA-SA fallback operations complete the population.

Following Stage 1, AHGA-SA stores the unique feature counts of the best solutions found up to this point, represented by $C^{★}$. In Stage 2, HTPI reinitializes the population based on these target counts. For each target count $c\in C^{★}$, candidate masks are constructed by prioritizing Head features up to the target count and then filling the remaining positions through importance-weighted Tail sampling. Completion operations are used again, if needed, to reach the desired population size.

Algorithm 1 summarizes HTPI initialization and reinitialization within AHGA-SA.

**Algorithm 1** HTPI-AHGA-SA initialization and reinitialization procedure
**Input:** standard AHGA-SA search inputs and validation-accuracy fitness evaluator, sorted feature-importance vector $w=(w_{1},\ldots ,w_{d})$ for the *d* preselected candidate features, population size *N*, cumulative-importance threshold θ (default 0.85), head-size bounds $\left[H_{\min},H_{\max}\right]$, Stage-1 maximum Tail budget $M_{T}$, tail-weight power *p* (default 1.0), and small probability floor ε (default $10^{-12}$)**Output:** best binary feature mask $x_{\mathrm{best}}$
1:**procedure** HeadTailPartition($w,\theta ,H_{\min},H_{\max},M_{T}$)2:    $q_{j}\leftarrow \left(\sum_{i\leq j}w_{i}\right)/\left(\sum_{i}w_{i}\right)$    for j=1,…,d3:    $h\leftarrow \min\{\;j:q_{j}\geq \theta \;\}$4:    $h\leftarrow \mathrm{clip}(h,\;H_{\min},\;H_{\max})$;   h←clip(h,1,d)5:    H←{1,…,h};   T←{h+1,…,d}6:    $M_{T}^{\prime }\leftarrow \min(M_{T},\;|T|)$7:    **return** $H,\;T,\;M_{T}^{\prime }$8:
**end procedure**


 

9:**function** WeightedTailSample(*k*)10:    **if** k≤0 **or** T=⌀ **then**11:        **return** ⌀12:    **end if**13:    k←min(k,|T|)14:    $\pi_{i}\leftarrow \max\;\left(w_{i}^{\;p},\;\varepsilon \right)$ for each i∈T15:    $\pi_{i}\leftarrow \pi_{i}/\sum_{j\in T}\pi_{j}$16:    **return** *k* features sampled from T *without replacement* with probabilities $\left\{\pi_{i}\right\}$17:
**end function**


 

18:**procedure** Stage1-HTPI-Init19:    P←⌀20:    **Seed** 1 (Head-only): x←0; $x_{i}\leftarrow 1\;\forall i\in H$; add x to *P* if distinct21:    **Seed** 2 (Head + maximum Tail budget): x←0; $x_{i}\leftarrow 1\;\forall i\in H$; $x_{i}\leftarrow 1\;\forall i\in$ WeightedTailSample $\left(M_{T}^{\prime }\right)$; add x to *P* if distinct22:    attempts←0;   max_attempts←100N23:    **while** |P|<N **and** attempts<max_attempts **do**24:        attempts←attempts+125:        x←0;   $x_{i}\leftarrow 1\;\forall i\in H$26:        **if** $M_{T}^{\prime }>0$ **then** $k∼\mathrm{Uniform}\{1,\ldots ,M_{T}^{\prime }\}$ **else** k←027:        $x_{i}\leftarrow 1\;\forall i\in$ WeightedTailSample(k)28:        add x to *P* if distinct29:    **end while**30:    **if** |P|<N **then**31:        modify donor masks through bit-flip mutation for up to 50N attempts, adding only distinct nonempty masks32:        **if** |P|<N **then** add distinct uniformly generated nonempty masks for up to 50N attempts33:        **if** |P|<N **then** duplicate existing masks until |P|=N34:    **end if**35:    **return** *P*36:
**end procedure**


 

37:**procedure** Stage2-HTPI-Reinit($C^{★}$)38:    $C^{★}\leftarrow$ Distinct$\left(C^{★}\right)$39:    P←⌀;   $n_{c}\leftarrow \max\;\left(1,\left\lfloor N/|C^{★}|\right\rfloor \right)$40:    **for all** target count $c\in C^{★}$ **do**41:        $P_{c}\leftarrow ⌀$;   attempts←042:        **while** $|P_{c}|<n_{c}$ **and** $\mathrm{attempts}<100n_{c}$ **do**43:           attempts←attempts+1;   x←0;   S←⌀44:           **if** 1<c≤|H| **then**45:               S←{1,…,c−1}∪{onerandomi∈H∖{1,…,c−1}}46:           **else**47:               S←{1,…,min(|H|,c)}48:           **end if**49:           r←c−|S|;   S←S ∪  WeightedTailSample (r)50:           **if** |S|<c **then** add c−|S| features drawn uniformly from (H∪T)∖S51:           $x_{i}\leftarrow 1\;\forall i\in S$;   add x to $P_{c}$ if distinct52:        **end while**53:        **if** $|P_{c}|<n_{c}$ **and** $|P_{c}|>0$ **then**54:           modify donor masks through bit-flip mutation for up to $50n_{c}$ attempts, adding only distinct nonempty masks55:        **end if**56:        **if** $|P_{c}|<n_{c}$ **then** generate $n_{c}-\left|P_{c}\right|$ uniformly random nonempty masks and add distinct masks57:        **if** $|P_{c}|<n_{c}$ **then** duplicate masks from $P_{c}$ until $|P_{c}|=n_{c}$58:        append all masks in $P_{c}$ to *P*59:    **end for**60:    truncate *P* to *N*; **if** |P|<N **then** complete *P* with masks from Stage1-HTPI-Init61:    **return** *P*62:
**end procedure**


**Integration with AHGA-SA:**
The GA, SA, fitness-evaluation, and duplicate-removal operations are inherited unchanged from Original AHGA-SA.
63:$\left(H,T,M_{T}^{\prime }\right)\leftarrow$ HeadTailPartition$\left(w,\theta ,H_{\min},H_{\max},M_{T}\right)$ after XGBoost preselection; reuse the resulting partition across the corresponding dataset runs.64:**Stage 1:** initialize P← Stage1-HTPI-Init and then run the unchanged Stage-1 GA-SA search.65:**Stage 2:** collect the distinct feature counts $C^{★}$ of the best Stage-1 solutions, reinitialize P← Stage2-HTPI-Reinit$\left(C^{★}\right)$, and then run the unchanged Stage-2 GA-SA search.66:**Return** $x_{\mathrm{best}}$.

**Fallback completion.** The attempt limits and fallback sequence are specified in Algorithm 1. Stage-2 masks produced by the mutation, random, or duplicate fallback were not required to preserve the corresponding target count. Across the 400 main HTPI-AHGA-SA runs, the mutation-based fallback supplied 85 individuals in total, while the random and duplicate fallbacks were not triggered.

### 3.4. Experimental Setup and Model Evaluation

The shared computational environment and experimental configuration are summarized in Table 2 and Table 3, respectively.

The XGBoost preselection model was fitted using only the internal-training rows of each processed dataset, and the corresponding Head–Tail partition was derived from the resulting feature-importance ranking. The preprocessing statistics and transformations used to construct those matrices followed the dataset-specific procedures described in the preceding subsection. These included the inherited pre-split transformations for the three legacy datasets and the external-training-derived medians and structural decisions for the five added datasets.

The Original AHGA-SA search settings were retained unchanged to preserve direct comparability. The HTPI settings were fixed during the design phase, applied unchanged across all eight datasets, and are not claimed to be globally optimal.

Both configurations used the same processed data, splits, classifiers, population size, search operators, two-stage structure, hardware, and thread allocation, and followed the same nominal schedule of 24 candidate evaluations per run. The realized count was identical in 351 of the 400 paired runs, lower for HTPI-AHGA-SA in 48 runs, and higher by one evaluation in one run. Overall, HTPI-AHGA-SA used 9552 evaluations compared with 9599 for Original AHGA-SA. The realized shortfalls arose from the shared duplicate-removal step inherited unchanged from Original AHGA-SA: duplicate candidate masks are removed before fitness evaluation and are therefore not reevaluated. Relative to the nominal 1200 evaluations per dataset, HTPI-AHGA-SA was short by 48 evaluations and Original AHGA-SA by one. Of the 48 HTPI shortfalls, 46 occurred on Edge-IIoTset (ML) and IoMT-TrafficData, the two datasets with the smallest candidate pools (d=11). A descriptive equal-budget sensitivity check was also conducted. The only algorithmic modification between the two search configurations was population construction during Stage-1 initialization and Stage-2 reinitialization.

**Detection model.** The same XGBoost-based detection model as in the original AHGA-SA study was used to isolate the effect of HTPI from changes in the downstream classifier. For each dataset and run, the final XGBoost classifier was fitted on the external training partition using the selected feature subset and evaluated on the corresponding external test partition.
**Fitness function.** For each candidate feature mask x, an XGBoost classifier was trained on the internal training subset using the selected features, and validation accuracy was used as the fitness value:
  $$F\left(x\right)={\mathrm{Accuracy}}_{\mathrm{val}}\left(x\right).$$
A mask containing no selected features was assigned a fitness value of −∞ and excluded from the evaluated population. Subset size was not included in the fitness value and was used only to break ties between candidates with equal validation accuracy.
**Performance metrics.** Predictive performance was evaluated using accuracy, macro-averaged precision, macro-averaged recall (Recall Macro), macro-averaged F1 score (F1 Macro), specificity, false-positive rate (FPR), false-negative rate (FNR), and Matthews correlation coefficient (MCC), consistent with the original AHGA-SA protocol [17,56]. The attack class was treated as the positive class for FPR, FNR, attack-class recall, and Average Precision (AP). Because all tasks were binary, attack-class recall equals 1−FNR, while specificity corresponds to normal-class recall. Attack-class AP was additionally computed from predicted probabilities on the external test partition to provide a threshold-independent assessment of precision–recall performance. Final XGBoost models were reconstructed from the stored selected feature subsets using the original data splits and classifier settings. An initial reproduction gate covering five seeds for each method–dataset combination reproduced F1 Macro, Recall Macro, accuracy, and MCC to machine precision. A subsequent full check reproduced F1 Macro, Recall Macro, and MCC across all 800 reconstructed runs within an absolute tolerance of $10^{-6}$. AP was then computed for the 50 seed-paired runs of the two configurations on each dataset.
**Cross-run stability protocol.** Both configurations were run 50 times using paired seeds 1–50. The run seed initialized the NumPy random generators governing population construction, importance-weighted Tail sampling, fallback completion, and the unchanged GA and SA operators. The data splits and all XGBoost classifier instances remained fixed at random_state=10 and random_state=42, respectively. Dataset-level external train/test counts and class balances are reported in Table A3 in Appendix C. For each dataset, the preprocessing outputs and XGBoost preselection ranking were held fixed; the Head–Tail partition was also fixed across HTPI runs. The reported values therefore quantify algorithmic cross-run stability under a fixed data realization, not stability under resampling, temporal drift, or alternative partitions.

Let $Z_{ij}=1$ when candidate feature *j* is selected in run *i* and $Z_{ij}=0$ otherwise. Nogueira’s size-corrected stability was computed as [57]

$$\hat{\Phi }=1-\frac{d^{-1}\sum_{j=1}^{d}s_{j}^{2}}{\left(\bar{k}/d\right)\left(1-\bar{k}/d\right)}.$$

Here, M=50 is the number of runs, $s_{j}^{2}$ is the unbiased sample variance of $\left\{Z_{1j},\ldots ,Z_{Mj}\right\}$, $\bar{k}$ is the mean selected-subset size, and *d* is the complete XGBoost-preselected candidate-pool size. The dataset-specific values of *d* are reported in Table 1; candidate features not selected in any run remain included in the normalization.

The 95% CIs were estimated using a leave-one-run-out jackknife. Stability differences were tested using two-sided paired *t*-tests on the leave-one-run-out jackknife pseudo-value differences paired by seed. Cohen’s $d_{z}$ was computed as the mean paired pseudo-value difference divided by its standard deviation, and Holm correction was applied across the eight datasets. CI separation was retained as descriptive support.

Two post hoc diagnostics of final-subset variation were also computed. The unique-subset ratio is the number of distinct selected subsets divided by the 50 runs. The mean pairwise symmetric difference is the mean number of features belonging to only one of two selected subsets across all run pairs. These measures describe variation in the final selected subsets across runs and do not measure population diversity within a run. Nogueira stability and the three primary predictive metrics (F1 Macro, MCC, and Recall Macro) were also recomputed descriptively on seed pairs for which both configurations used the same number of candidate evaluations.

**Statistical and practical comparison.** Predictive metrics were compared over the 50 paired seeds using two-sided Wilcoxon signed-rank tests with Pratt handling of zero paired differences. Holm correction was applied separately to each metric across the eight datasets, and matched-pairs rank-biserial correlations were used as effect sizes. Selected-feature counts were analyzed using the same paired Wilcoxon and Holm-correction procedure. The ±0.003 F1 Macro margin, equivalent to 0.3 percentage points, was specified during the design phase and is treated as a study-specific practical tolerance rather than a universal IDS standard. The criterion |ΔF1|<0.003 is used descriptively, while formal equivalence was assessed using the paired two one-sided tests (TOST) procedure with the same margin and 90% CIs. Paired TOST was computed from the paired seed-level F1 Macro differences using the corresponding two one-sided paired *t*-tests, with the reported raw $p_{\mathrm{TOST}}$ taken as the larger of the two one-sided *p*-values. These paired tests characterize run-to-run algorithmic differences under the fixed data partition and do not quantify uncertainty over alternative samples or data splits.
**Targeted component ablation.** A targeted sequential ablation was conducted on IoTID20, WUSTL-EHMS, and Edge-IIoTset (ML) to examine the contributions of the HTPI population-construction components. Four conditions were evaluated using the same fixed splits, preselection rankings, classifier settings, search operators, and 50 paired seeds. These were Original AHGA-SA with random initialization; basic Head–Tail initialization with uniform Tail sampling; basic Head–Tail initialization with importance-weighted Tail sampling; and Full HTPI with importance-weighted Tail sampling and Stage-2-aware reinitialization. Both basic Head–Tail conditions omitted Stage-2-aware count constraints. The sequential contrasts were interpreted descriptively as the incremental contributions of basic Head–Tail initialization, importance-weighted Tail sampling, and Stage-2-aware reinitialization. This analysis was targeted rather than a full factorial ablation.
**Post hoc explainability analyses.** Post hoc XAI was conducted on IoTID20, WUSTL-EHMS, and Edge-IIoTset (ML), representing general, medical, and industrial IoT domains. The analyses interpret the selected subsets after the FS search and do not affect initialization, fitness evaluation, parameter selection, or subset selection.

## 4. Results

This section compares Original AHGA-SA and HTPI-AHGA-SA across the eight IoT-oriented IDS datasets in terms of selected-feature count, runtime, cross-run stability, predictive performance, supplementary attack-class AP and recall, tail-weight power sensitivity, and targeted component ablation.

### 4.1. Feature-Subset Size, Runtime, and Cross-Run Stability

The mean number of selected features, mean per-run runtime, and Nogueira’s size-corrected stability are reported in Table 4 for both configurations. Runtime is the mean measured per-run execution time from population initialization through final model fitting and test-metric computation; data loading, preprocessing, and XGBoost preselection are excluded. The 95% leave-one-run-out jackknife CIs are reported for Nogueira stability. CI separation indicates that the lower bound of the HTPI-AHGA-SA CI is above the upper bound of the Original AHGA-SA CI.

Runtime differences in Table 4 are reported descriptively because no significance test was applied to runtime. Formal paired inference for Nogueira stability is reported in Table 5. Stability remains significantly higher under HTPI-AHGA-SA on all eight datasets after Holm correction, with relative gains ranging from 138.0% to 491.1% and Cohen’s $d_{z}$ values ranging from 0.564 to 1.844.

**Figure 2:**
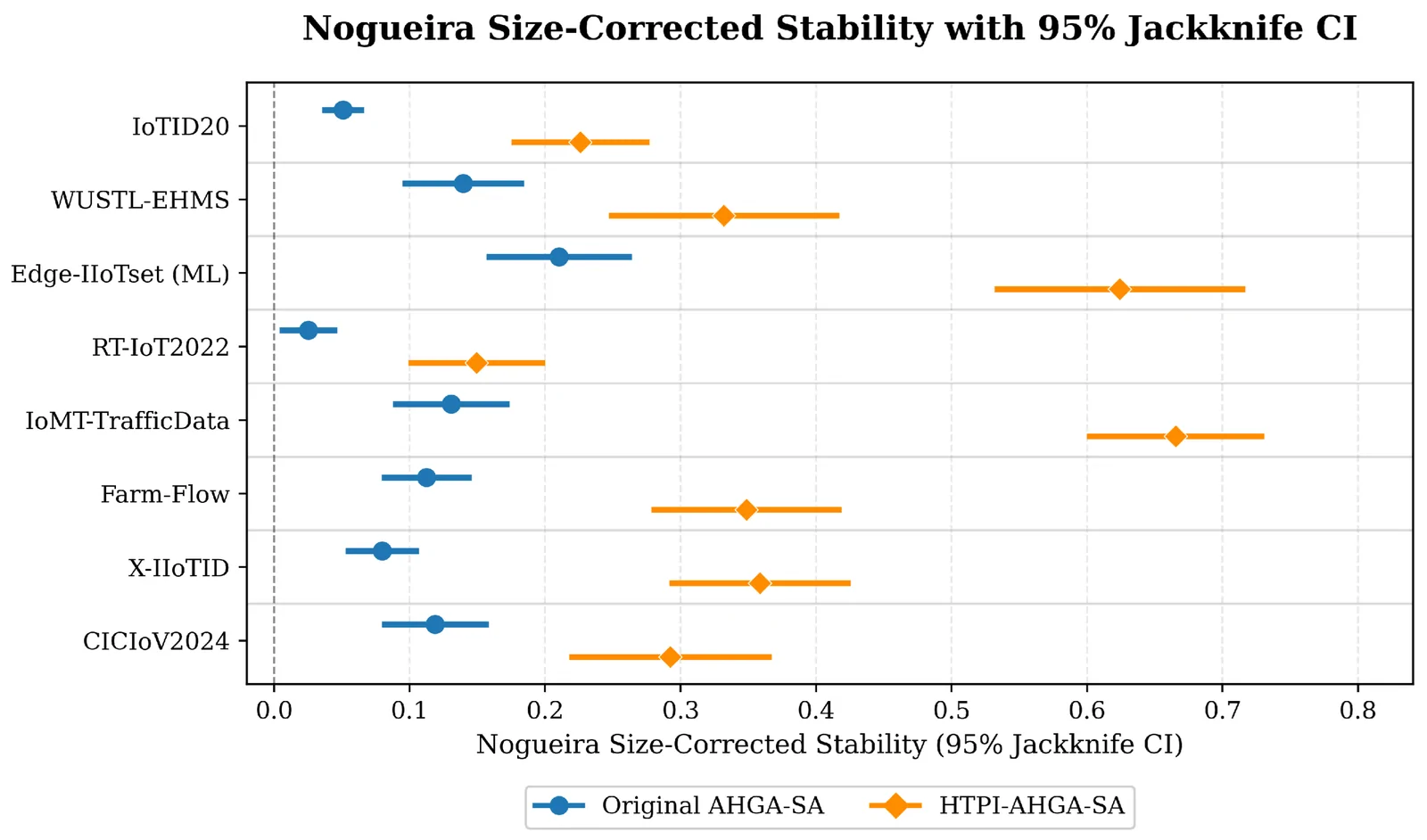
Nogueira stability with 95% leave-one-run-out jackknife CIs across the eight datasets; the dashed vertical line marks zero stability.

Figure 2 visualizes the dataset-level Nogueira stability estimates and 95% CIs reported in Table 4.

**Figure 3:**
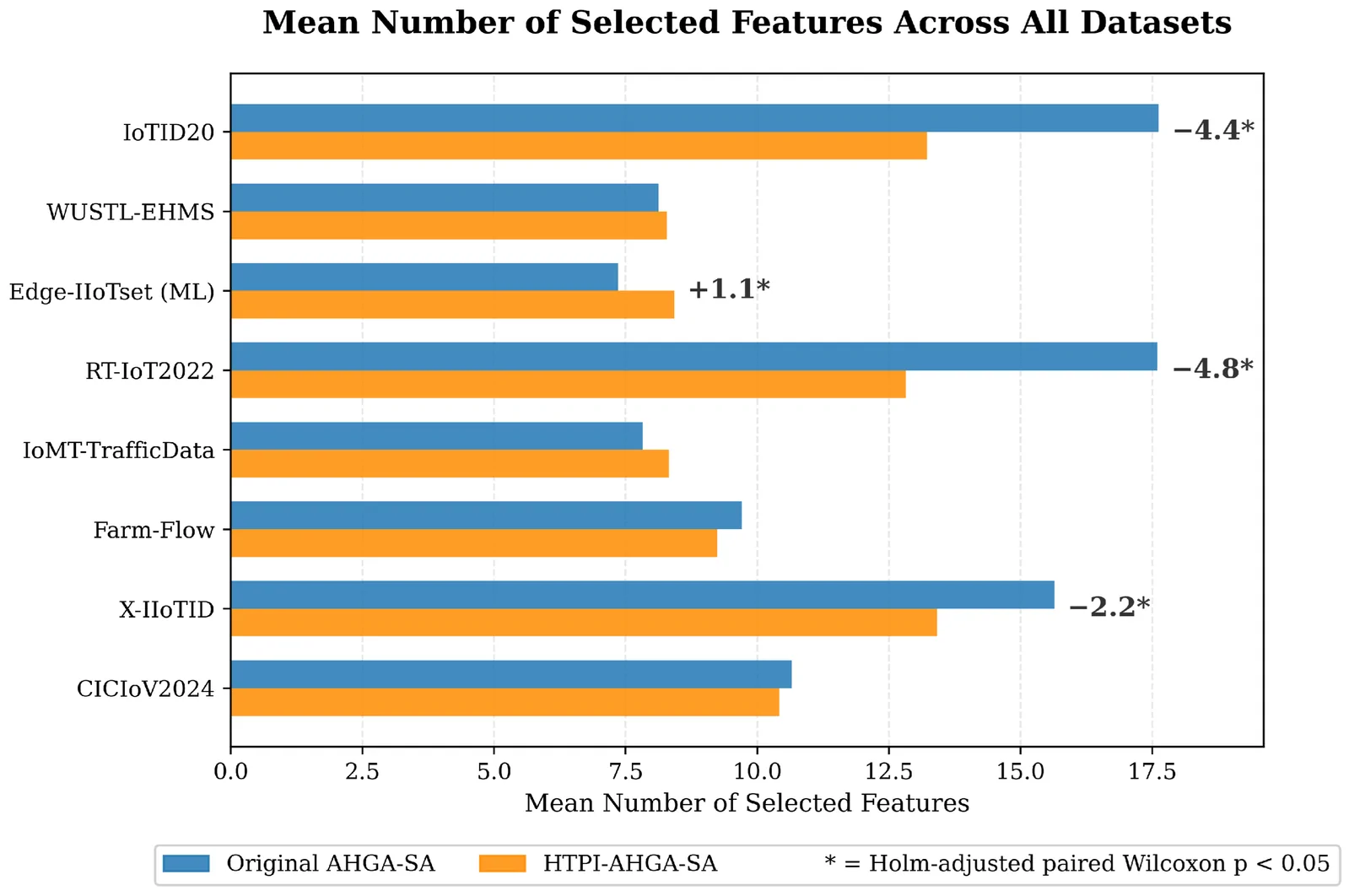
Mean selected-feature count across the eight datasets. Annotated values are mean differences (HTPI-AHGA-SA minus Original AHGA-SA); an asterisk indicates a Holm-adjusted paired Wilcoxon p<0.05.

Figure 3 compares the mean selected-feature counts of the two configurations. HTPI-AHGA-SA selects fewer features on five datasets and slightly more features on three datasets, indicating dataset-dependent subset-size behavior.

**Figure 4:**
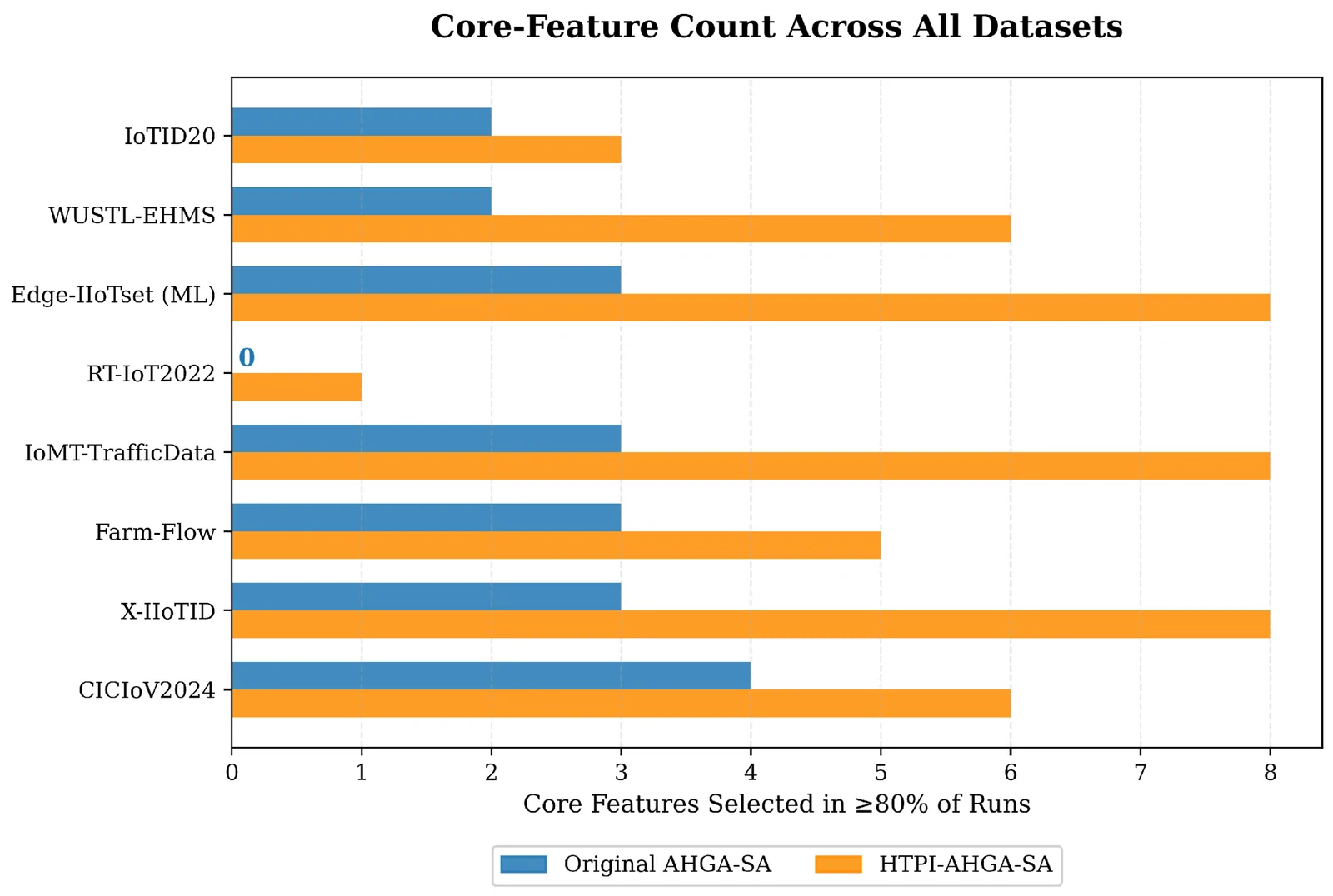
Core-feature count across the eight datasets. Core features are selected in ≥80% of runs.

The number of core features selected in at least 80% of the 50 runs is reported in Figure 4. HTPI-AHGA-SA yields more core features on all eight datasets, with the largest increases on Edge-IIoTset (ML), IoMT-TrafficData, and X-IIoTID.

**Figure 5:**
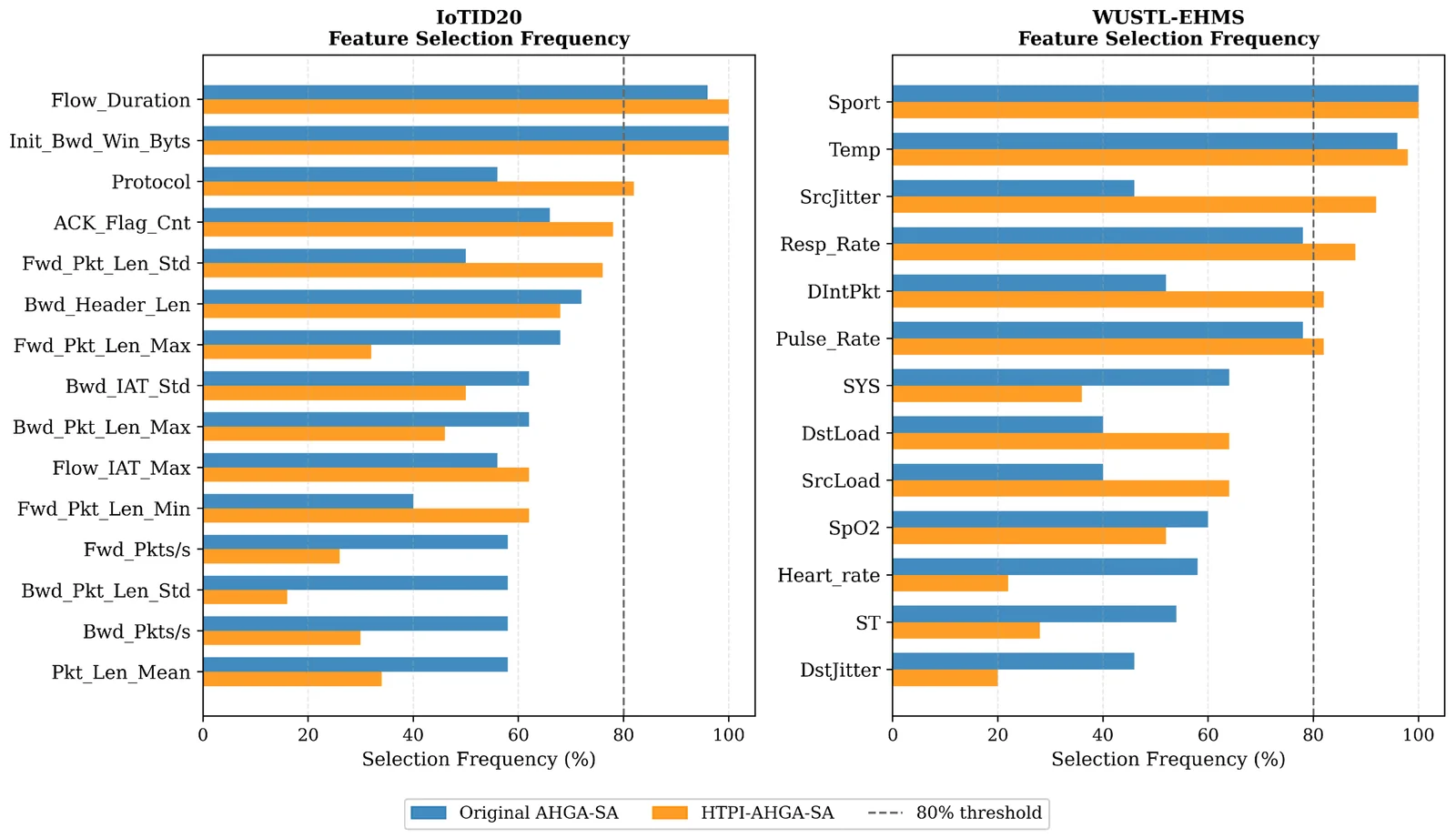
Per-feature selection frequency for IoTID20 and WUSTL-EHMS. Each panel shows up to 15 features from the union selected by either method, ordered by the higher method-specific frequency; the dashed line marks the 80% core-feature threshold.

Figure 5 shows dataset-dependent changes in feature-selection frequency. For example, Protocol on IoTID20 and SrcJitter on WUSTL-EHMS are selected more frequently under HTPI-AHGA-SA.

### 4.2. Predictive Performance

**Figure 6:**
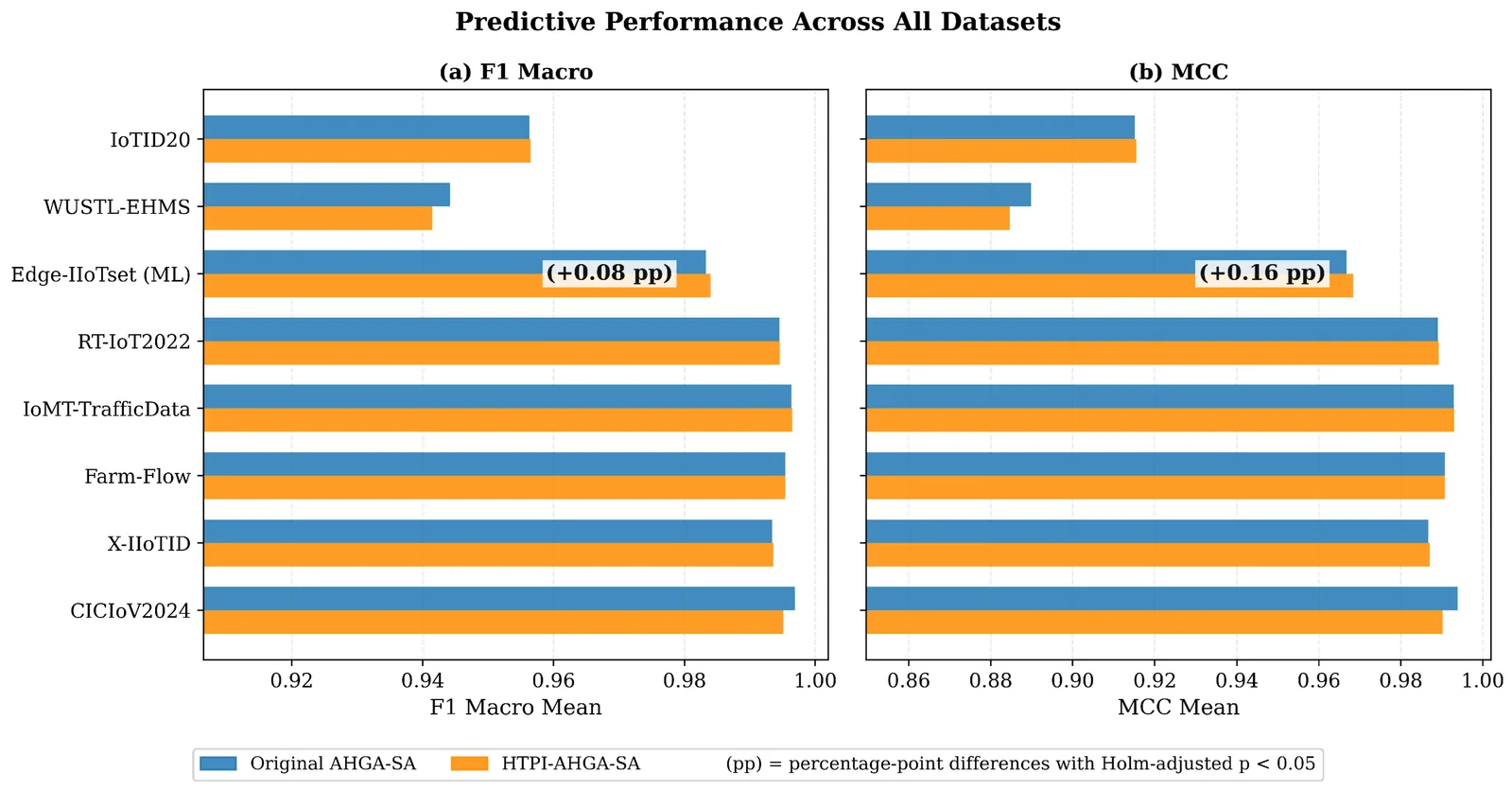
Mean F1 Macro and MCC across the eight datasets. Annotations show percentage-point differences (HTPI-AHGA-SA minus Original AHGA-SA) that remain significant after within-metric Holm correction.

Figure 6 provides a visual overview of mean F1 Macro and MCC across the eight datasets. Table 6 reports the exact mean predictive performance values and paired mean differences, including Recall Macro, while Table 7 presents the corresponding effect sizes, Wilcoxon tests, Holm-adjusted *p*-values, and paired TOST outcomes for F1 Macro.

Across the eight datasets, all absolute F1 Macro differences are below 0.003. Paired TOST supports formal equivalence for six datasets; equivalence is not supported for WUSTL-EHMS or CICIoV2024. After Holm correction, the F1 Macro and MCC differences remain significant only for Edge-IIoTset (ML), both in favor of HTPI-AHGA-SA. Recall Macro remains significant for Edge-IIoTset (ML) in favor of HTPI-AHGA-SA and for CICIoV2024 in favor of Original AHGA-SA. Additional predictive performance metrics are reported in Table A4 in Appendix D.

Supplementary attack-class AP and recall results are reported in Table A5 in Appendix D. After Holm correction, AP is lower under HTPI-AHGA-SA on WUSTL-EHMS and CICIoV2024, higher on IoMT-TrafficData, and statistically lower but numerically almost unchanged on RT-IoT2022. No significant AP difference remains on the other four datasets, and attack-class recall does not differ significantly on any dataset after Holm correction. These results highlight dataset-specific probability-ranking trade-offs and distinguish threshold-independent AP from the threshold-dependent predictive metrics reported in Table 6 and statistically compared in Table 7.

### 4.3. Sensitivity of HTPI to the Tail-Weight Power

**Figure 7:**
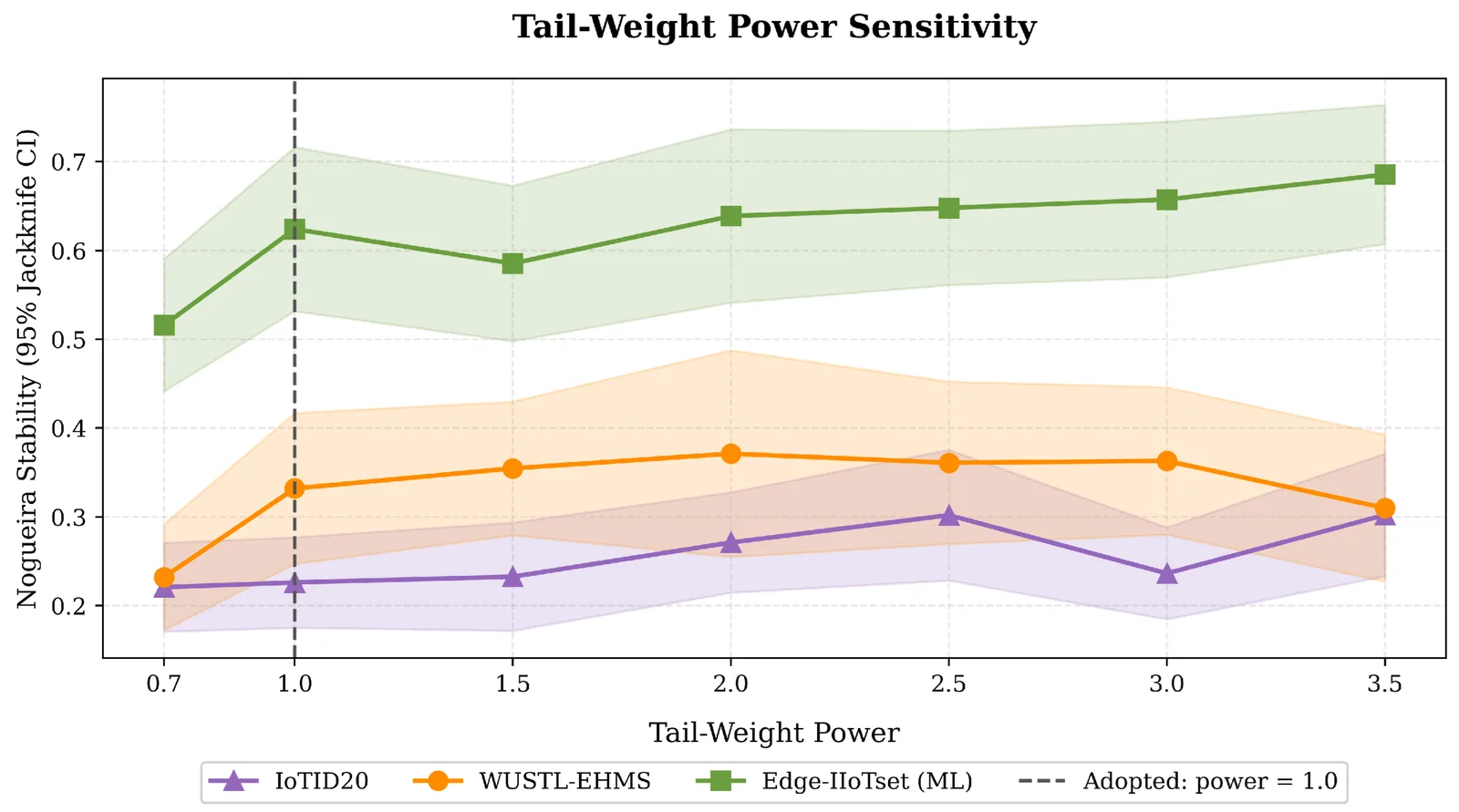
Nogueira stability across tail-weight powers with 95% jackknife CIs; shaded areas indicate the corresponding 95% CIs, and the dashed line marks the adopted p=1.0.

Figure 7 and Table A6 in Appendix E report the sensitivity of Nogueira stability and ΔF1 Macro to the tail-weight power *p* on IoTID20, WUSTL-EHMS, and Edge-IIoTset (ML). Each power value was evaluated over the same 50 seeds, with all other algorithm settings and data partitions held fixed. The adopted value p=1.0 corresponds to linear importance weighting.

The effect of the tail-weight power is dataset-dependent and non-monotonic. Across all tested values, IoTID20 and Edge-IIoTset (ML) retained absolute F1 Macro differences below the study-specific descriptive threshold of 0.003, although their stability estimates varied across the sweep. WUSTL-EHMS was more sensitive: p=1.0 and p=1.5 remained below the threshold, whereas p=0.7 and p≥2.0 exceeded it.

### 4.4. Targeted Component Ablation

Table 8 reports the targeted sequential ablation results on IoTID20, WUSTL-EHMS, and Edge-IIoTset (ML). All three Head–Tail-based variants achieve higher point estimates of Nogueira stability than Original AHGA-SA across the three datasets. These nine comparisons are descriptive, and no significance claim is made for the individual component contrasts.

The ablation results indicate that no single HTPI component explains the stability gain uniformly across datasets. Basic Head–Tail initialization provides the largest contribution on IoTID20, whereas importance-weighted Tail sampling provides the largest contribution on WUSTL-EHMS and Edge-IIoTset (ML). The effect of Stage-2-aware reinitialization is smaller and dataset-dependent: it increases the stability estimate on IoTID20 but does not further increase it on WUSTL-EHMS or Edge-IIoTset (ML). Full HTPI nevertheless achieves the highest or tied mean F1 Macro among the Head–Tail-based variants on all three datasets, indicating that the complete design balances cross-run stability with predictive behavior rather than maximizing stability alone.

## 5. Post Hoc XAI Analysis

Two post hoc analyses are reported on the three original AHGA-SA benchmark datasets: PI-based cross-run consistency and retraining-based group sufficiency. IoTID20, WUSTL-EHMS, and Edge-IIoTset (ML) represent general, medical, and industrial IoT domains, respectively, and provide representative rather than exhaustive XAI evidence. Both analyses were conducted only after the FS search and did not affect HTPI initialization, fitness evaluation, parameter selection, or feature-subset selection.

For both post hoc analyses, models were fitted on the external training partition. For IoTID20 and Edge-IIoTset (ML), a fixed class-stratified subsample of 10,000 observations was drawn once from the external test partition by sampling each class without replacement in proportion to its test-partition frequency using numpy.random.default_rng(10). The full WUSTL-EHMS external test partition of 3264 observations was used. The same evaluation sample was reused across all runs and both configurations. The size of 10,000 observations was an operational computational choice and was not tuned.

### 5.1. PI-Based Cross-Run Consistency

PI assesses a trained model’s reliance on a feature by randomly permuting that feature and measuring the resulting change in model performance. This technique was first proposed for random forests by Breiman [58] and later extended to a model-agnostic measure of model reliance by Fisher et al. [59]. For each run, the final XGBoost detection model was reconstructed using that run’s selected subset, and PI was computed as the mean decrease in F1 Macro across five permutation repeats using random_state=10 and n_jobs=16. PI was not fed back into FS or parameter tuning. Because the evaluation sample was derived from the external test partition, the PI results are treated as descriptive post hoc evidence.

PI vectors were aligned to the common preselected feature pool, assigning zero to features absent from a run’s selected subset. The resulting zero-filled Spearman measure therefore reflects both selection consistency and PI-ranking consistency. To reduce the influence of non-selection, conditional Spearman correlations were also computed for run pairs sharing at least three selected features. Because run pairs are not independent, these pairwise distributions are reported descriptively and are not used for hypothesis testing.

Top-*k* Jaccard measures the mean pairwise Jaccard overlap between the highest-PI feature sets across runs, using k=7 for IoTID20 and k=5 for WUSTL-EHMS and Edge-IIoTset (ML). Within each run, features are ranked only within that run’s selected subset; when fewer than *k* features are selected, the complete selected subset is used. Thus, unselected features are never eligible for a Top-*k* set. For each run, Core-PI coverage is the fraction of positive PI assigned to features selected in at least 80% of the runs, and the reported value is the mean across runs. The values of *k* and the 80% threshold are operational reporting choices rather than tuned parameters.

**Figure 8:**
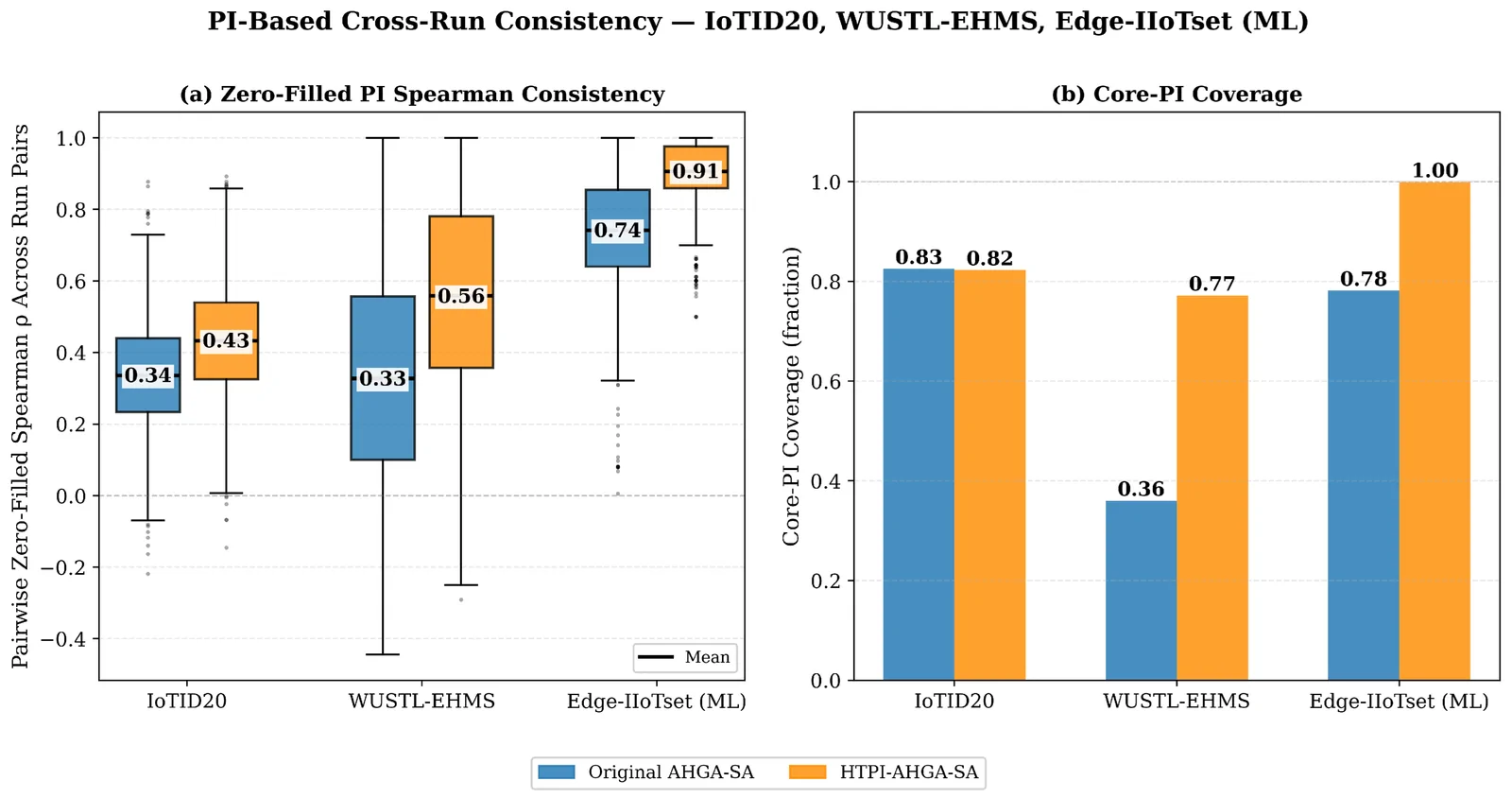
Post hoc permutation importance (PI)-based cross-run consistency. Panel (**a**) shows pairwise zero-filled Spearman correlations across run pairs; numeric annotations indicate means. Panel (**b**) reports Core-PI coverage using the adopted 80% core-feature threshold.

The PI-based cross-run consistency results are summarized in Figure 8, with the adopted-setting metrics and reporting-choice sensitivity results reported in Table A7 and Table A8, respectively, in Appendix F.

HTPI-AHGA-SA achieves higher mean zero-filled Spearman correlation and mean Top-*k* Jaccard overlap on all three datasets. Under the conditional analysis, it remains higher on IoTID20 and Edge-IIoTset (ML), while the values differ only slightly on WUSTL-EHMS (0.842 versus 0.838). As a sensitivity check, Top-*k* Jaccard was higher under HTPI-AHGA-SA in all 12 dataset–*k* combinations for k∈{3,5,7,10}. Core-PI coverage was higher in seven of the nine dataset–threshold combinations for thresholds of 70%, 80%, and 90%; the two exceptions were IoTID20 at the 80% and 90% thresholds, where the values were approximately 0.826 for Original AHGA-SA and 0.823 for HTPI-AHGA-SA.

### 5.2. Retraining-Based Group Sufficiency Analysis

Removal-based explanations measure the importance of a feature or feature group by removing, masking, or withholding it and observing the resulting change in model behavior. This principle underlies several XAI approaches, including SHapley Additive exPlanations (SHAP) [60], Local Interpretable Model-agnostic Explanations (LIME) [61], permutation-based tests, and feature-ablation methods, which have been discussed within a common removal-based framework [62]. Feature ablation is implemented in interpretability toolkits such as Captum [63], while grouped feature ablation has also been highlighted in recent XAI surveys [64]. The present study adopts the broader removal principle through a retraining-based group sufficiency analysis rather than fixed-model feature ablation.

For each run, three feature-set conditions were constructed: Full, Head-only, and Tail-only. Head-only and Tail-only therefore refer to the selected portions of the two partitions, not to their complete feature pools. For each condition, an XGBoost classifier was retrained from scratch on the external training set using the same dataset-specific settings reported in Table 3, so only the input feature set differed. Each retrained model was evaluated on the fixed XAI evaluation sample described above. All nonempty conditions were evaluated, including single-feature conditions. Empty conditions were excluded because a classifier cannot be fitted without input features. Two Original AHGA-SA Head-only conditions on WUSTL-EHMS were empty and were the only exclusions among the 900 run–condition combinations.

**Figure 9:**
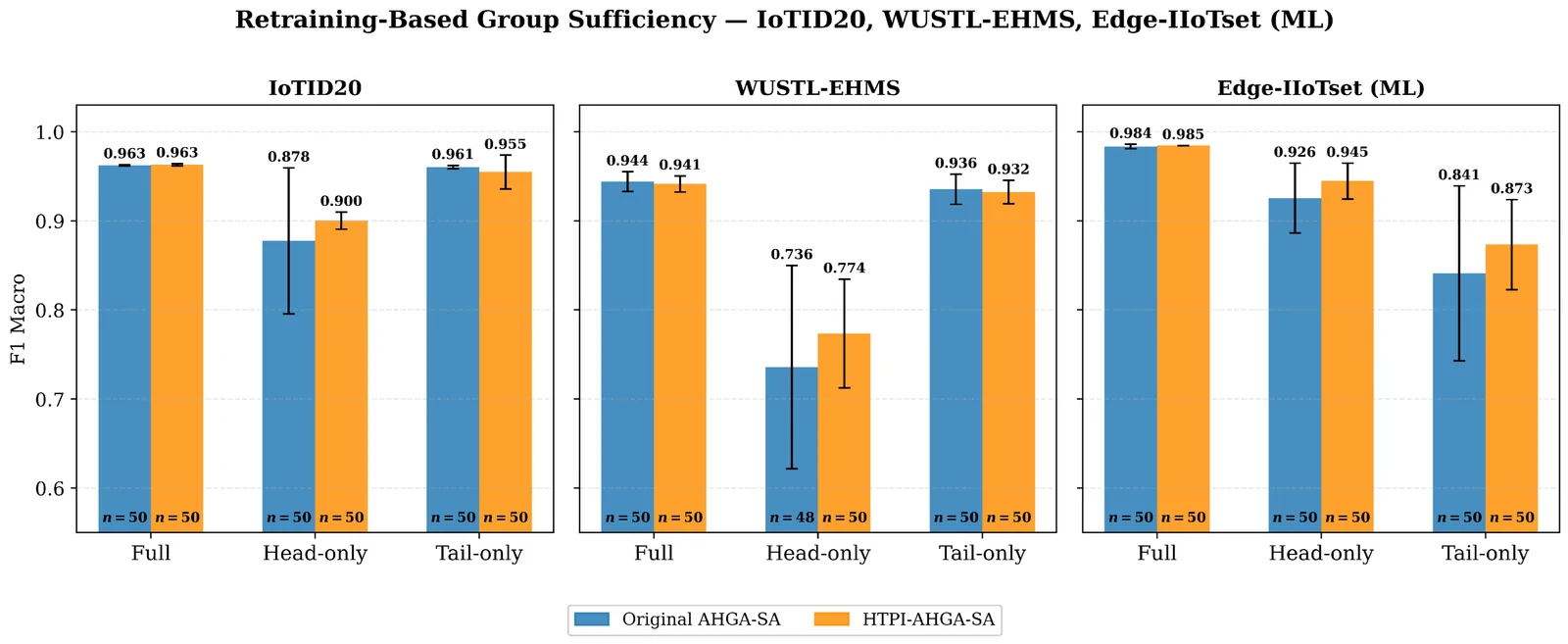
Retraining-based group sufficiency on the representative datasets. Bars show mean F1 Macro, and error bars show ±1 sample standard deviation; *n* denotes the number of valid runs.

Figure 9 summarizes retraining-based group sufficiency on the three representative datasets, with detailed numerical results reported in Table A9 in Appendix G.

Head-only subsets retain measurable predictive utility across all three datasets. Tail-only subsets remain close to the Full condition on IoTID20 and WUSTL-EHMS, whereas the corresponding gaps are substantially larger on Edge-IIoTset (ML). The Full condition has the highest mean F1 Macro in all six method–dataset combinations. These results provide complementary descriptive evidence that the selected Head and Tail portions can each retain predictive utility under retraining.

## 6. Discussion

HTPI-AHGA-SA improves FS stability across all eight datasets, with the paired stability tests remaining significant after Holm correction and the 95% leave-one-run-out jackknife CIs completely separated. Predictive performance differences remain generally small but dataset-dependent. Because subset-size changes are dataset-dependent, HTPI’s primary observed effect is reduced run-to-run variability under the fixed split rather than improved predictive performance or universal compactness.

Improved FS stability can be valuable even when predictive performance changes only slightly because it provides more consistent feature preferences across repeated stochastic runs [10,16,57]. In deployed IDS pipelines, consistently selected subsets may reduce changes to feature-extraction, validation, and explanation workflows. In IoT environments, predictable feature requirements may also simplify model comparison, maintenance, auditing, and incident analysis. These operational effects were not evaluated in a live deployment.

Although all absolute F1 Macro differences are below the predefined descriptive criterion, paired TOST supports formal equivalence for only six of the eight datasets; equivalence is not supported for WUSTL-EHMS or CICIoV2024. The latter also illustrates the need to interpret stability gains alongside security-relevant trade-offs: FNR increases from 0.56% to 1.34%, corresponding to a decrease in attack-class recall from 99.44% to 98.66%, while F1 Macro and MCC decrease by 0.19 and 0.37 percentage points, respectively. For CICIoV2024, the Recall Macro difference remains significant after Holm correction, whereas the attack-class recall difference does not. After Holm correction, AP is lower on WUSTL-EHMS and CICIoV2024, lower by a numerically negligible amount on RT-IoT2022, and higher on IoMT-TrafficData. Stability gains should therefore be interpreted together with Table 6 and Table 7 and the supplementary security metrics in Table A4 and Table A5.

The sensitivity and targeted ablation analyses indicate that HTPI’s behavior is dataset-dependent. Across the tail-weight power sweep, IoTID20 and Edge-IIoTset (ML) remained within the predefined F1 Macro criterion, whereas WUSTL-EHMS exceeded it under several settings; at the adopted linear setting p=1.0, HTPI-AHGA-SA retained higher Nogueira stability on all three datasets while remaining within the criterion. The ablation results likewise show that no single component dominates uniformly: basic Head–Tail initialization contributes most on IoTID20, importance-weighted Tail sampling on WUSTL-EHMS and Edge-IIoTset (ML), and Stage-2-aware reinitialization has a smaller, dataset-dependent effect. Full HTPI nevertheless achieves the highest or tied mean F1 Macro among the Head–Tail variants, so the adopted weighting and complete design should be viewed as an evaluated stability–performance compromise rather than as universally optimal settings.

The post hoc XAI analyses provide complementary descriptive evidence: PI-based consistency is generally higher under HTPI-AHGA-SA, and both selected Head and Tail portions retain measurable predictive utility under retraining. The Full condition remains strongest across all six method–dataset combinations, while Tail-only performance is close to Full on IoTID20 and WUSTL-EHMS but shows a larger gap on Edge-IIoTset (ML). These analyses did not affect HTPI initialization, fitness evaluation, parameter selection, or subset selection.

Post hoc diagnostics of the returned subsets and the equal-budget sensitivity check are reported in Table A10 in Appendix H. The unique-subset ratio is lower under HTPI-AHGA-SA on six datasets, and the mean pairwise symmetric difference is lower on all eight; these patterns are treated as descriptive measures of cross-run subset variation rather than independent performance benefits. In the equal-budget sensitivity check, HTPI-AHGA-SA retains higher Nogueira stability on all eight datasets. The directions of the F1 Macro, MCC, and Recall Macro differences are unchanged across all eight datasets, and all absolute F1 Macro differences remain below the study-specific 0.003 descriptive criterion. Because the equal-budget restriction is post hoc, these comparisons are interpreted descriptively rather than as causal effects of evaluation budget.

HTPI intentionally biases initialization toward the XGBoost ranking. Tail sampling and the unchanged crossover, mutation, and SA operations retain opportunities to explore lower-ranked features. However, ranking errors or redundant Head features may reduce the exposure of useful Tail combinations. Because population diversity and convergence were not measured directly, the results support improved cross-run repeatability rather than unrestricted exploration or attainment of a global optimum.

The conclusions are limited to the evaluated HTPI-AHGA-SA configuration and do not establish superiority over all initialization strategies. The fixed-split protocol measures algorithmic cross-run stability, not stability under alternative samples, temporal partitions, or data drift. For the five added datasets, preprocessing statistics and structural decisions were derived from the complete external training partition, including its internal-validation rows. This limits the strict independence of internal-validation preprocessing, although the external test partition remained excluded from those statistics and decisions. The same processed matrices and fitted transformations were used for both configurations. The evaluation relies on XGBoost-based ranking and does not include a full factorial component ablation, factorial parameter analysis, or direct diversity and convergence measurements. Some datasets in the final evaluation also informed the design-phase parameter choices; therefore, the sensitivity results should not be interpreted as fully external parameter validation.

For the three legacy benchmark datasets, numerical median imputation and categorical encoding followed the original AHGA-SA implementation and preceded the external split, which preserved direct comparability with the published baseline. These operations were based on feature distributions and used no target-label information at any stage. Within each dataset, both configurations used the same processed matrices, so any resulting effect is common to the paired comparison and is not expected to differentially favor either method. The Nogueira stability calculation is also based on the resulting binary feature-selection matrix rather than on test labels or test predictions. We nevertheless note conservatively that the retained legacy preprocessing may make the absolute predictive estimates optimistic for these three datasets. The search fitness uses validation accuracy, which may be sensitive to class imbalance; macro-averaged and attack-class metrics are assessed only after feature selection. Finally, the post hoc XAI analysis is limited to three representative datasets and uses the external test realization or a fixed test subsample, as applicable, for descriptive interpretation.

## 7. Conclusions and Future Work

This study evaluated HTPI as an incremental extension of AHGA-SA that replaces only its population initialization and reinitialization steps. Across all eight IoT-oriented datasets, HTPI-AHGA-SA achieved significantly higher Nogueira stability after Holm correction, with non-overlapping 95% leave-one-run-out jackknife CIs in all cases. All absolute differences in dataset-level mean F1 Macro remained below 0.003, although formal equivalence at this margin was supported for only six datasets, and dataset-specific security-metric trade-offs remained, particularly on CICIoV2024.

On three representative datasets, the post hoc XAI analyses indicated generally more reproducible selection-and-importance patterns under HTPI-AHGA-SA and measurable predictive utility in both the selected Head and Tail portions under retraining. These analyses were descriptive and were conducted only after feature selection.

The findings are limited to the evaluated HTPI-AHGA-SA configuration, IoT-oriented binary intrusion detection, and XGBoost-based preselection; stability was assessed under a fixed data partition for each dataset. Future work should compare HTPI with alternative population-initialization strategies and evaluate it on non-IoT cybersecurity datasets and high-dimensional classification problems, as well as with alternative ranking methods, classifiers, multiclass attack settings, and data-splitting protocols.

## Figures and Tables

**Table 1:** Summary of the datasets used in the experimental evaluation.

Dataset	Ref.	IoT Domain	Samples	Features (Initial/Processed)	Candidate Pool (*d*)	Class Distribution (N/A, %)
IoTID20	[40]	General	625,783	83/65	31	6.4/93.6
WUSTL-EHMS	[42]	Medical	16,318	43/36	13	87.5/12.5
Edge-IIoTset (ML)	[46]	Industrial	157,800	61/42	11	15.4/84.6
RT-IoT2022	[50]	Smart Home	123,117	84/81	33	10.2/89.8
IoMT-TrafficData	[44]	Medical	585,743	31/30	11	51.2/48.8
Farm-Flow	[52]	Agricultural	564,626	30/29	15	50.8/49.2
X-IIoTID	[48]	Industrial	820,834	65/54	26	51.3/48.7
CICIoV2024	[54]	Vehicular	1,408,219	153/75	17	86.9/13.1

Ref.: reference; IoT: Internet of Things; N: normal; A: attack. Initial features are counted after excluding the target and alternate-label columns; Processed denotes the feature count after the removals listed in Table A1; *d* is the fixed size of the XGBoost-preselected candidate pool containing only features with non-zero importance; the same pool is used by both methods.

**Table 2:** Hardware and software setup.

Component	Specification
Processor	Intel Core i9-14900K
Memory	192 GB DDR5
Storage	2 TB Samsung 990 PRO
Operating System	Windows 11 Pro
Programming environment	Python 3.14.3; NumPy 2.1.0; pandas 2.2.3; scikit-learn 1.6.1; SciPy 1.15.2; XGBoost 3.0.0

**Table 3:** Experimental configuration and parameter settings.

Configuration Category	Parameter	Value
Experimental setup	Train/test split	80%/20%; shuffled; random_state=10
	Internal train/validation split	80%/20% from the training set; shuffled; stratified; random_state=10
	Evaluation runs per method	50 runs using seeds 1–50
	Stability confidence interval estimation	Leave-one-run-out jackknife over 50 runs
Original AHGA-SA parameters	Population size (*N*)	4
	Fixed search stages ($S_{\max}$)	2
	Tournament selection size (*S*)	2
	Crossover probability ($C_{r}$)	0.7
	Mutation probability ($M_{r}$)	0.2
	Initial temperature ($T_{\mathrm{init}}$)	95
	Cooling rate (α)	0.90
Proposed HTPI parameters	Head selection method	Cumulative importance
	Cumulative-importance threshold (θ)	0.85
	Minimum/maximum head size ($H_{\min}/H_{\max}$)	4/6
	Tail sampling strategy	Importance-weighted, without replacement
	Tail-weight power (*p*)	1.0
	Probability floor (ε)	$10^{-12}$
	Stage-1 maximum Tail budget ($M_{T}$)	4, fixed
XGBoost configuration	Preselection	XGBoost classifier (n_estimators=2)
	Fitness and final detection models	XGBoost classifier (n_estimators = *)
	Classification objective	binary:logistic
	Preselection importance type	normalized gain
	XGBoost eval_metric	logloss
	Random state	random_state=42; reapplied to each classifier instance
	Tree method	hist
	Processor threads	n_jobs=16
	All remaining XGBoost parameters	Library defaults for the reported XGBoost version; no additional parameter search

* n_estimators: 150 for WUSTL-EHMS; 75 for Edge-IIoTset (ML) and RT-IoT2022; 25 for IoTID20, IoMT-TrafficData, Farm-Flow, and X-IIoTID; and 15 for CICIoV2024.

**Table 4:** Main feature-count, runtime, and stability results.

Dataset	Feat. (O/HT)	RT (ms) (O/HT)	Nogueira (O/HT)	O 95% CI	HT 95% CI	CI Sep.
IoTID20	17.62/13.22	4233/3414	0.051/0.226	[0.035, 0.066]	[0.175, 0.277]	YES
WUSTL-EHMS	8.12/8.28	1444/1518	0.140/0.332	[0.095, 0.184]	[0.247, 0.417]	YES
Edge-IIoTset (ML)	7.36/8.42	1843/1851	0.210/0.624	[0.157, 0.264]	[0.532, 0.717]	YES
RT-IoT2022	17.60/12.82	1961/1675	0.025/0.149	[0.004, 0.047]	[0.099, 0.200]	YES
IoMT-TrafficData	7.82/8.32	2250/2288	0.131/0.665	[0.088, 0.174]	[0.600, 0.731]	YES
Farm-Flow	9.70/9.24	2745/2587	0.112/0.349	[0.079, 0.146]	[0.279, 0.419]	YES
X-IIoTID	15.64/13.42	5406/4562	0.080/0.359	[0.053, 0.107]	[0.292, 0.426]	YES
CICIoV2024	10.66/10.42	4324/4335	0.119/0.292	[0.079, 0.158]	[0.217, 0.367]	YES

Feat.: mean number of selected features; O: Original AHGA-SA; HT: HTPI-AHGA-SA; RT: mean runtime per run; CI: 95% leave-one-run-out jackknife CI; CI Sep.: CI separation. Nogueira stability is normalized by the complete preselected candidate-pool size *d* reported in Table 1.

**Table 5:** Formal paired inference for the difference in Nogueira stability.

Dataset	ΔΦ	Relative Gain	95% CI for ΔΦ	$d_{z}$	Raw *p*	$p_{\mathrm{Holm}}$
IoTID20	0.1752	344.7%	[0.1245, 0.2252]	0.963	$1.32\times 10^{-8}$	$6.61\times 10^{-8}$
WUSTL-EHMS	0.1925	138.0%	[0.1042, 0.2797]	0.606	$8.42\times 10^{-5}$	$1.68\times 10^{-4}$
Edge-IIoTset (ML)	0.4137	196.7%	[0.3146, 0.5091]	1.174	$6.71\times 10^{-11}$	$4.69\times 10^{-10}$
RT-IoT2022	0.1241	491.1%	[0.0721, 0.1765]	0.660	$2.39\times 10^{-5}$	$7.18\times 10^{-5}$
IoMT-TrafficData	0.5347	409.2%	[0.4530, 0.6133]	1.844	$1.51\times 10^{-17}$	$1.21\times 10^{-16}$
Farm-Flow	0.2363	210.2%	[0.1622, 0.3100]	0.886	$9.13\times 10^{-8}$	$3.65\times 10^{-7}$
X-IIoTID	0.2787	349.3%	[0.2042, 0.3533]	1.036	$2.07\times 10^{-9}$	$1.24\times 10^{-8}$
CICIoV2024	0.1735	146.0%	[0.0884, 0.2593]	0.564	$2.21\times 10^{-4}$	$2.21\times 10^{-4}$

ΔΦ: HTPI-AHGA-SA minus Original AHGA-SA; relative gain: ΔΦ/Φ_Original_ × 100; CI: 95% confidence interval for the paired stability difference; *d_z_*: paired standardized effect size. Raw p-values are from two-sided paired *t*-tests on the jackknife pseudo-value differences. Holm correction was applied across the eight datasets.

**Table 6:** Predictive performance of Original AHGA-SA and HTPI-AHGA-SA.

Dataset	F1 Macro (O/HT)	ΔF1	MCC (O/HT)	ΔMCC	Recall Macro (O/HT)	ΔRecall
IoTID20	0.9563/0.9565	+0.0002	0.9151/0.9155	+0.0003	0.9282/0.9286	+0.0003
WUSTL-EHMS	0.9442/0.9415	−0.0027	0.8898/0.8846	−0.0052	0.9260/0.9224	−0.0036
Edge-IIoTset (ML)	0.9833/0.9840	+0.0008	0.9668/0.9684	+0.0016	0.9900/0.9929	+0.0030
RT-IoT2022	0.9945/0.9946	+0.0001	0.9891/0.9893	+0.0002	0.9921/0.9922	+0.0001
IoMT-TrafficData	0.9964/0.9965	+0.0001	0.9929/0.9930	+0.0002	0.9964/0.9965	+0.0001
Farm-Flow	0.9954/0.9954	+0.0000	0.9908/0.9908	+0.0000	0.9953/0.9953	+0.0000
X-IIoTID	0.9934/0.9936	+0.0002	0.9867/0.9871	+0.0004	0.9933/0.9935	+0.0002
CICIoV2024	0.9969/0.9951	−0.0019	0.9939/0.9902	−0.0037	0.9968/0.9930	−0.0038

O: Original AHGA-SA; HT: HTPI-AHGA-SA; Δ: HT − O. Mean differences were computed from the unrounded means across the 50 paired runs.

**Table 7:** Paired statistical tests, effect sizes, and F1 Macro equivalence assessment.

Dataset	Metric	$r_{\mathrm{rb}}$	*p*	$p_{\mathrm{Holm}}$	$p_{\mathrm{TOST}}$	90% CI of ΔF1	F1 Equivalence
IoTID20	F1 Macro	+0.376	0.0201	0.1006	<0.001	[0.00004, 0.00032]	Supported
	MCC	+0.357	0.0275	0.1102	–	–	–
	Recall Macro	+0.396	0.0148	0.0887	–	–	–
WUSTL-EHMS	F1 Macro	−0.405	0.0119	0.0831	0.433	[−0.00604, 0.00072]	Not supported
	MCC	−0.402	0.0126	0.0880	–	–	–
	Recall Macro	−0.385	0.0171	0.0887	–	–	–
Edge-IIoTset (ML)	F1 Macro	+0.667	0.0002	0.0014	<0.001	[0.00020, 0.00134]	Supported
	MCC	+0.690	0.0001	0.0010	–	–	–
	Recall Macro	+0.718	<0.0001	0.0006	–	–	–
RT-IoT2022	F1 Macro	+0.167	0.3344	0.6687	<0.001	[−0.00003, 0.00019]	Supported
	MCC	+0.179	0.3016	0.6032	–	–	–
	Recall Macro	+0.112	0.5240	1.0000	–	–	–
IoMT-TrafficData	F1 Macro	+0.369	0.0230	0.1006	<0.001	[0.00003, 0.00014]	Supported
	MCC	+0.385	0.0178	0.0898	–	–	–
	Recall Macro	+0.360	0.0267	0.1069	–	–	–
Farm-Flow	F1 Macro	+0.056	0.7282	0.7282	<0.001	[−0.00002, 0.00004]	Supported
	MCC	+0.039	0.8093	0.8093	–	–	–
	Recall Macro	+0.047	0.7721	1.0000	–	–	–
X-IIoTID	F1 Macro	+0.222	0.1750	0.5250	<0.001	[−0.00007, 0.00047]	Supported
	MCC	+0.224	0.1719	0.5157	–	–	–
	Recall Macro	+0.217	0.1844	0.5533	–	–	–
CICIoV2024	F1 Macro	−0.400	0.0154	0.0922	0.144	[−0.00364, −0.00010]	Not supported
	MCC	−0.402	0.0150	0.0898	–	–	–
	Recall Macro	−0.488	0.0033	0.0233	–	–	–

MCC: Matthews correlation coefficient; CI: confidence interval; TOST: two one-sided tests; $r_{\mathrm{rb}}$: matched-pairs rank-biserial correlation; positive values favor HTPI-AHGA-SA. *p*: raw Wilcoxon paired signed-rank *p*-value over the 50 paired seeds. $p_{\mathrm{Holm}}$: Holm-adjusted within each metric across the eight datasets. $p_{\mathrm{TOST}}$: raw paired TOST *p*-value for F1 Macro at the ±0.003 margin, with the corresponding 90% CI. Holm adjustment of the eight TOST *p*-values preserves the same six equivalence outcomes. Holm-adjusted values were computed from the unrounded raw *p*-values.

**Table 8:** Targeted sequential ablation of HTPI components.

Dataset	Condition	Nogueira	95% CI	F1 Macro	Feat.	Mean Evals.
IoTID20	Original	0.0508	[0.0355, 0.0662]	0.9563±0.0004	17.62	24.00
	Basic Head–Tail, uniform Tail	0.1790	[0.1428, 0.2152]	0.9560±0.0020	13.08	23.00
	Basic Head–Tail, weighted Tail	0.1756	[0.1435, 0.2076]	0.9564±0.0005	13.26	22.98
	Full HTPI	0.2261	[0.1751, 0.2770]	0.9565±0.0005	13.22	24.00
WUSTL-EHMS	Original	0.1395	[0.0947, 0.1844]	0.9442±0.0111	8.12	24.00
	Basic Head–Tail, uniform Tail	0.1649	[0.1033, 0.2266]	0.9345±0.0204	8.32	23.00
	Basic Head–Tail, weighted Tail	0.3779	[0.2804, 0.4753]	0.9371±0.0167	8.00	22.96
	Full HTPI	0.3320	[0.2470, 0.4170]	0.9415±0.0090	8.28	23.98
Edge-IIoTset (ML)	Original	0.2103	[0.1567, 0.2640]	0.9833±0.0024	7.36	24.00
	Basic Head–Tail, uniform Tail	0.4037	[0.3086, 0.4989]	0.9839±0.0005	8.92	22.82
	Basic Head–Tail, weighted Tail	0.6503	[0.5451, 0.7556]	0.9840±0.0003	8.50	22.06
	Full HTPI	0.6241	[0.5316, 0.7165]	0.9840±0.0003	8.42	23.40

Feat.: mean selected-feature count; Mean evals.: mean realized number of candidate evaluations. Basic Head–Tail conditions retain Head–Tail initialization in both stages but omit the Stage-2-aware elite-count constraint; F1 Macro values are reported as mean ± sample standard deviation across the 50 runs.

## Data Availability

The datasets used in this study are publicly available from their original sources: IoTID20 [40], WUSTL-EHMS [42], Edge-IIoTset (ML) [46], RT-IoT2022 [50], IoMT-TrafficData [44], Farm-Flow [52], X-IIoTID [48], and CICIoV2024 [54]. No new datasets were generated or collected in this study.

## Appendix A. Removed Feature Columns

**Table A1:** Removed feature columns per dataset.

Dataset	Count	Removed Features
**IoTID20**	18	Flow_ID, Src_IP, Dst_IP, Src_Port, Dst_Port, Timestamp, Active_Max, Bwd_IAT_Max, Bwd_Seg_Size_Avg, Fwd_IAT_Max, Fwd_Seg_Size_Avg, Idle_Max, PSH_Flag_Cnt, Pkt_Size_Avg, Subflow_Bwd_Byts, Subflow_Bwd_Pkts, Subflow_Fwd_Byts, Subflow_Fwd_Pkts
**WUSTL-EHMS**	7	Dir, Flgs, SrcAddr, DstAddr, SrcMac, DstMac, Packet_num
**Edge-IIoTset (ML)**	19	frame.time, ip.src_host, ip.dst_host, arp.src.proto_ipv4, arp.dst.proto_ipv4, http.file_data, http.request.full_uri, icmp.transmit_timestamp, http.request.uri.query, tcp.options, tcp.payload, tcp.srcport, tcp.dstport, udp.port, mqtt.msg, mqtt.topic, mqtt.protoname, dns.qry.name.len, mqtt.conack.flags
**RT-IoT2022**	3	no, id.orig_p, bwd_URG_flag_count
**IoMT-TrafficData**	1	traffic
**Farm-Flow**	1	traffic
**X-IIoTID**	11	Date, Timestamp, Scr_IP, Scr_port, Des_IP, Des_port, anomaly_alert, OSSEC_alert, OSSEC_alert_level, Bad_checksum, is_SYN_with_RST
**CICIoV2024**	78	ID0, ID1, ID2, ID3, ID4, ID5, DATA_00, DATA_01, DATA_02, DATA_03, DATA_04, DATA_05, DATA_06, DATA_07, DATA_08, DATA_10, DATA_11, DATA_12, DATA_13, DATA_14
		DATA_15, DATA_16, DATA_17, DATA_18, DATA_20, DATA_21, DATA_22, DATA_23, DATA_24, DATA_25, DATA_26, DATA_27, DATA_28, DATA_30, DATA_31, DATA_32, DATA_33, DATA_34, DATA_35, DATA_36
		DATA_37, DATA_38, DATA_40, DATA_41, DATA_42, DATA_43, DATA_44, DATA_45, DATA_46, DATA_47, DATA_48, DATA_50, DATA_51, DATA_52, DATA_53, DATA_54, DATA_55, DATA_56, DATA_57, DATA_58
		DATA_60, DATA_61, DATA_62, DATA_63, DATA_64, DATA_65, DATA_66, DATA_67, DATA_68, DATA_70, DATA_71, DATA_72, DATA_73, DATA_74, DATA_75, DATA_76, DATA_77, DATA_78

## Appendix B. XGBoost Preselection Feature-Importance Rankings

**Table A2:** Top-10 XGBoost preselection feature-importance rankings.

Dataset	No.	Feature	Imp. (%)	Group	Dataset	No.	Feature	Imp. (%)	Group
**IoTID20**	1	ACK_Flag_Cnt	85.74	H	**WUSTL-EHMS**	1	SrcLoad	71.20	H
	2	Fwd_Pkt_Len_Std	6.12	H		2	DIntPkt	10.04	H
	3	Flow_Duration	2.83	H		3	SrcJitter	6.16	H
	4	Protocol	1.15	H		4	Resp_Rate	2.77	H
	5	Fwd_Pkt_Len_Min	1.05	T		5	DstLoad	2.37	T
	6	Init_Bwd_Win_Byts	1.00	T		6	Sport	2.34	T
	7	Flow_IAT_Max	0.54	T		7	Pulse_Rate	2.14	T
	8	Bwd_Pkt_Len_Min	0.34	T		8	ST	0.81	T
	9	Bwd_Header_Len	0.25	T		9	SpO2	0.80	T
	10	Bwd_IAT_Std	0.21	T		10	DstJitter	0.50	T
**Edge-IIoTset (ML)**	1	mqtt.hdrflags	25.03	H	**RT-IoT2022**	1	flow_iat.min	69.73	H
	2	tcp.len	22.66	H		2	flow_RST_flag_count	13.36	H
	3	tcp.seq	12.58	H		3	service	4.29	H
	4	http.request.version	11.08	H		4	flow_pkts_payload.min	3.84	H
	5	tcp.flags	10.31	H		5	bwd_subflow_bytes	2.18	T
	6	tcp.connection.synack	8.49	H		6	flow_CWR_flag_count	1.10	T
	7	tcp.ack	5.85	T		7	fwd_pkts_per_sec	0.83	T
	8	tcp.ack_raw	3.19	T		8	proto	0.79	T
	9	udp.time_delta	0.47	T		9	fwd_pkts_payload.min	0.76	T
	10	tcp.checksum	0.33	T		10	flow_pkts_payload.max	0.40	T
**IoMT-TrafficData**	1	total_data_pkts	95.97	H	**Farm-Flow**	1	orig_pkts	92.35	H
	2	fwd_bwd_payload_avg_diff	1.93	H		2	resp_pkts	3.98	H
	3	total_bytes	0.79	H		3	orig_ip_bytes	1.16	H
	4	history_responder	0.51	H		4	bwd_header_size_max	0.63	H
	5	payload_ratio	0.46	T		5	fwd_pkts_per_sec	0.60	T
	6	flow_payload_range	0.16	T		6	bwd_pkts_payload.tot	0.38	T
	7	service	0.12	T		7	flow_iat.tot	0.26	T
	8	pkts_unidirectional_traffic_0	0.04	T		8	bwd_pkts_payload.avg	0.18	T
	9	header_size_ratio	0.00	T		9	bwd_header_size_tot	0.16	T
	10	header_size_diff	0.00	T		10	down_up_ratio	0.14	T
**X-IIoTID**	1	is_with_payload	42.08	H	**CICIoV2024**	1	DATA_310	52.20	H
	2	Avg_num_cswch/s	28.63	H		2	DATA_316	10.86	H
	3	Login_attempt	11.42	H		3	DATA_710	8.35	H
	4	Conn_state	5.31	H		4	ID13	7.22	H
	5	read_write_physical.process	3.18	T		5	DATA_611	6.60	H
	6	is_syn_only	3.11	T		6	DATA_713	3.72	T
	7	Service	3.00	T		7	ID10	3.19	T
	8	Avg_nice_time	0.83	T		8	ID11	1.64	T
	9	Avg_num_Proc/s	0.59	T		9	DATA_59	1.56	T
	10	Duration	0.53	T		10	ID8	1.43	T

Imp.: XGBoost gain importance, normalized over the candidate pool and reported as a percentage rounded to two decimal places; entries shown as 0.00 are strictly positive values below 0.005%. H: Head group; T: Tail group.

## Appendix C. Train/Test Split Details

**Table A3:** Train/test split details per dataset.

Dataset	Train Total (N/A)	Test Total (N/A)	Train Balance (N/A, %)	Test Balance (N/A, %)
IoTID20	500,626 (32,168/468,458)	125,157 (7905/117,252)	6.4/93.6	6.3/93.7
WUSTL-EHMS	13,054 (11,372/1682)	3264 (2900/364)	87.1/12.9	88.8/11.2
Edge-IIoTset (ML)	126,240 (19,471/106,769)	31,560 (4830/26,730)	15.4/84.6	15.3/84.7
RT-IoT2022	98,493 (10,135/88,358)	24,624 (2372/22,252)	10.3/89.7	9.6/90.4
IoMT-TrafficData	468,594 (240,172/228,422)	117,149 (59,828/57,321)	51.3/48.7	51.1/48.9
Farm-Flow	451,700 (229,455/222,245)	112,926 (57,199/55,727)	50.8/49.2	50.7/49.3
X-IIoTID	656,667 (337,112/319,555)	164,167 (84,305/79,862)	51.3/48.7	51.4/48.6
CICIoV2024	1,126,575 (979,209/147,366)	281,644 (244,528/37,116)	86.9/13.1	86.8/13.2

N: normal; A: attack.

## Appendix D. Additional Predictive Performance Metrics

**Table A4:** Extended predictive performance metrics.

Metric	Method	IoTID20	WUSTL-EHMS	Edge-IIoTset (ML)	RT-IoT2022	IoMT-TrafficData	Farm-Flow	X-IIoTID	CICIoV2024
Acc.	O	99.03%	97.88%	99.12%	99.81%	99.64%	99.54%	99.34%	99.86%
	HT	99.03%	97.78%	99.16%	99.81%	99.65%	99.54%	99.36%	99.78%
Prec.	O	98.89%	96.48%	97.70%	99.70%	99.65%	99.55%	99.34%	99.72%
	HT	98.89%	96.31%	97.56%	99.71%	99.66%	99.55%	99.36%	99.72%
Spec.	O	85.72%	99.40%	98.82%	98.47%	99.93%	99.97%	99.59%	99.92%
	HT	85.79%	99.37%	99.49%	98.48%	99.94%	99.97%	99.62%	99.95%
FPR	O	14.28%	0.60%	1.18%	1.53%	0.07%	0.03%	0.41%	0.08%
	HT	14.21%	0.63%	0.51%	1.52%	0.06%	0.03%	0.38%	0.05%
FNR	O	0.07%	14.20%	0.82%	0.05%	0.66%	0.91%	0.93%	0.56%
	HT	0.07%	14.88%	0.90%	0.04%	0.65%	0.91%	0.92%	1.34%
F1 Macro	O	95.63%	94.42%	98.33%	99.45%	99.64%	99.54%	99.34%	99.69%
	HT	95.65%	94.15%	98.40%	99.46%	99.65%	99.54%	99.36%	99.51%
MCC	O	91.51%	88.98%	96.68%	98.91%	99.29%	99.08%	98.67%	99.39%
	HT	91.55%	88.46%	96.84%	98.93%	99.30%	99.08%	98.71%	99.02%
Recall Macro	O	92.82%	92.60%	99.00%	99.21%	99.64%	99.53%	99.33%	99.68%
	HT	92.86%	92.24%	99.29%	99.22%	99.65%	99.53%	99.35%	99.30%

Acc.: accuracy; Prec.: macro-averaged precision; Spec.: specificity; FPR: false-positive rate; FNR: false-negative rate; MCC: Matthews correlation coefficient; O: Original AHGA-SA; HT: HTPI-AHGA-SA. Because attack is the positive class, specificity corresponds to normal-class recall. MCC values are scaled by 100. All reported values are means across the 50 runs.

**Table A5:** Attack-class average precision and attack-class recall results.

Dataset	AP (O/HT)	ΔAP	AP $p_{\mathrm{Holm}}$	Attack-Class Recall (O/HT)	Δ Attack-Class Recall	Attack-Class Recall $p_{\mathrm{Holm}}$
IoTID20	0.99963/0.99963	+0.00000	1.00000	0.99926/0.99926	−0.00001	1.00000
WUSTL-EHMS	0.96285/0.96011	−0.00274	0.01434	0.85802/0.85115	−0.00687	0.15680
Edge-IIoTset (ML)	0.99965/0.99970	+0.00005	0.66055	0.99177/0.99097	−0.00080	1.00000
RT-IoT2022	1.00000/0.99999	$-1.1\times 10^{-6}$	0.04837	0.99954/0.99955	+0.00002	0.78940
IoMT-TrafficData	0.99932/0.99937	+0.00005	0.00231	0.99344/0.99354	+0.00010	1.00000
Farm-Flow	0.99803/0.99798	−0.00005	0.30225	0.99093/0.99095	+0.00002	1.00000
X-IIoTID	0.99958/0.99958	−0.00001	1.00000	0.99073/0.99081	+0.00008	1.00000
CICIoV2024	0.99688/0.99278	−0.00410	0.01860	0.99441/0.98661	−0.00780	0.05874

O: Original AHGA-SA; HT: HTPI-AHGA-SA; AP: attack-class average precision calculated from predicted probabilities; Δ: HT − O. Attack-class recall is the attack-detection rate. $p_{\mathrm{Holm}}$: Holm-adjusted paired Wilcoxon *p*-value within each metric across the eight datasets. Differences were computed from unrounded means.

## Appendix E. Tail-Weight Power Sensitivity Results

**Table A6:** Tail-weight power sensitivity results using Nogueira stability and F1 Macro difference.

Dataset	Power	Nogueira	95% Jackknife CI	ΔF1 Macro (HTPI Mean ± SD)
IoTID20	0.7	0.221	[0.171, 0.271]	+0.0001 (0.9563 ± 0.0004)
	1.0	0.226	[0.175, 0.277]	+0.0002 (0.9565 ± 0.0005)
	1.5	0.233	[0.172, 0.294]	−0.0001 (0.9562 ± 0.0003)
	2.0	0.271	[0.215, 0.328]	+0.0001 (0.9563 ± 0.0004)
	2.5	0.302	[0.228, 0.376]	+0.0001 (0.9563 ± 0.0004)
	3.0	0.236	[0.185, 0.288]	+0.0001 (0.9564 ± 0.0004)
	3.5	0.302	[0.233, 0.371]	+0.0001 (0.9564 ± 0.0003)
WUSTL-EHMS	0.7	0.232	[0.172, 0.292]	−0.0056 * (0.9385 ± 0.0127)
	1.0	0.332	[0.247, 0.417]	−0.0027 (0.9415 ± 0.0090)
	1.5	0.355	[0.279, 0.430]	−0.0027 (0.9414 ± 0.0103)
	2.0	0.371	[0.255, 0.488]	−0.0052 * (0.9390 ± 0.0124)
	2.5	0.361	[0.270, 0.452]	−0.0054 * (0.9388 ± 0.0110)
	3.0	0.363	[0.280, 0.446]	−0.0033 * (0.9409 ± 0.0094)
	3.5	0.310	[0.227, 0.393]	−0.0067 * (0.9374 ± 0.0145)
Edge-IIoTset (ML)	0.7	0.516	[0.441, 0.590]	+0.0008 (0.9840 ± 0.0002)
	1.0	0.624	[0.532, 0.717]	+0.0008 (0.9840 ± 0.0003)
	1.5	0.585	[0.498, 0.673]	+0.0008 (0.9841 ± 0.0002)
	2.0	0.639	[0.541, 0.736]	+0.0008 (0.9841 ± 0.0002)
	2.5	0.648	[0.561, 0.735]	+0.0008 (0.9841 ± 0.0001)
	3.0	0.657	[0.570, 0.745]	+0.0008 (0.9841 ± 0.0002)
	3.5	0.686	[0.607, 0.764]	+0.0008 (0.9841 ± 0.0002)

CI: 95% leave-one-run-out jackknife CI; ΔF1 Macro: mean F1 Macro of HTPI-AHGA-SA minus Original AHGA-SA; * indicates |ΔF1|≥0.003 and does not denote statistical significance; SD: sample standard deviation.

## Appendix F. PI-Based Cross-Run Consistency and Sensitivity Results

**Table A7:** PI-based cross-run consistency metrics, including conditional analysis restricted to features selected in both runs of a pair.

Dataset	Method	Zero-Filled $\bar{\rho }$	Conditional $\bar{\rho }$	Pairs Used	Mean Shared Feat.	Top-*k* Jaccard	Core-PI Coverage
IoTID20	O	0.336	0.802	1225/1225	10.4	0.404	0.826
IoTID20	HT	0.433	0.933	1224/1225	7.4	0.446	0.823
WUSTL-EHMS	O	0.327	0.838	1216/1225	5.5	0.512	0.360
WUSTL-EHMS	HT	0.558	0.842	1225/1225	6.3	0.655	0.772
Edge-IIoTset (ML)	O	0.742	0.888	1223/1225	5.4	0.636	0.782
Edge-IIoTset (ML)	HT	0.906	0.927	1225/1225	7.7	0.779	0.999

O: Original AHGA-SA; HT: HTPI-AHGA-SA; PI: permutation importance. Zero-filled and conditional $\bar{\rho }$, Top-*k* Jaccard, and Core-PI coverage are defined in Section 5.1. Pairs used: pairs contributing to conditional $\bar{\rho }$ out of 502=1225; Mean shared feat.: mean shared-feature count among those pairs. The adopted values were k=7 for IoTID20 and k=5 for WUSTL-EHMS and Edge-IIoTset (ML); Core-PI coverage uses the adopted 80% threshold.

**Table A8:** Sensitivity of the post hoc PI metrics to the Top-*k* and core-threshold reporting choices. Values are reported as Original AHGA-SA/HTPI-AHGA-SA; adopted settings are shown in bold.

(a) Top-*k* Jaccard (Original/HTPI)				
**Dataset**	** k=3 **	** k=5 **	** k=7 **	** k=10 **
IoTID20	0.635/0.704	0.487/0.545	**0.404/0.446**	0.373/0.388
WUSTL-EHMS	0.534/0.681	**0.512/0.655**	0.488/0.590	0.517/0.623
Edge-IIoTset (ML)	0.802/0.837	**0.636/0.779**	0.602/0.830	0.589/0.841
**(b) Core-PI Coverage (Original/HTPI)**				
**Dataset**	**70%**	**80%**	**90%**	
IoTID20	0.864/0.884	**0.826/0.823**	0.826/0.823	
WUSTL-EHMS	0.474/0.772	**0.360/0.772**	0.360/0.371	
Edge-IIoTset (ML)	0.921/1.000	**0.782/0.999**	0.782/0.991	

Top-*k* Jaccard and Core-PI coverage are defined in Section 5.1. HTPI-AHGA-SA is higher in all 12 dataset–*k* combinations and in seven of the nine dataset–threshold combinations. The two exceptions are IoTID20 at the 80% and 90% thresholds.

## Appendix G. Retraining-Based Group Sufficiency Results

**Table A9:** Retraining-based group sufficiency results.

Dataset	Method	Condition	Valid Runs	Mean Features	F1 Macro, Mean (SD)	MCC, Mean (SD)	Recall Macro, Mean (SD)
**IoTID20**	O	Full	50	17.62	0.963 (0.001)	0.927 (0.002)	0.940 (0.001)
		Head-only	50	2.68	0.878 (0.082)	0.773 (0.161)	0.822 (0.068)
		Tail-only	50	14.94	0.961 (0.002)	0.923 (0.004)	0.937 (0.003)
	HT	Full	50	13.22	0.963 (0.001)	0.928 (0.002)	0.940 (0.002)
		Head-only	50	3.36	0.900 (0.010)	0.816 (0.017)	0.843 (0.012)
		Tail-only	50	9.86	0.955 (0.019)	0.912 (0.037)	0.935 (0.008)
**WUSTL-EHMS**	O	Full	50	8.12	0.944 (0.011)	0.890 (0.021)	0.926 (0.015)
		Head-only	48	2.25	0.736 (0.114)	0.513 (0.218)	0.693 (0.085)
		Tail-only	50	5.96	0.936 (0.017)	0.874 (0.032)	0.917 (0.025)
	HT	Full	50	8.28	0.941 (0.009)	0.885 (0.018)	0.922 (0.011)
		Head-only	50	3.26	0.774 (0.061)	0.579 (0.113)	0.725 (0.048)
		Tail-only	50	5.02	0.932 (0.013)	0.866 (0.025)	0.916 (0.018)
**Edge-IIoTset (ML)**	O	Full	50	7.36	0.984 (0.003)	0.968 (0.005)	0.990 (0.007)
		Head-only	50	4.52	0.926 (0.039)	0.857 (0.077)	0.918 (0.023)
		Tail-only	50	2.84	0.841 (0.098)	0.720 (0.150)	0.795 (0.085)
	HT	Full	50	8.42	0.985 (0.000)	0.970 (0.000)	0.993 (0.000)
		Head-only	50	5.72	0.945 (0.020)	0.895 (0.039)	0.924 (0.003)
		Tail-only	50	2.70	0.873 (0.051)	0.772 (0.080)	0.824 (0.045)

O: Original AHGA-SA; HT: HTPI-AHGA-SA; Full: complete selected subset; Head-only/Tail-only: selected features intersecting the Head/Tail partition; F1 Macro: macro-averaged F1 score; MCC: Matthews correlation coefficient; SD: sample standard deviation. All nonempty conditions were evaluated, including single-feature conditions. Two Original AHGA-SA Head-only conditions on WUSTL-EHMS contained no selected Head feature and were therefore excluded; this row retained 48 runs, while all other rows retained all 50 runs. Models were evaluated on the fixed XAI evaluation sample described in Section 5. Because IoTID20 and Edge-IIoTset (ML) used the fixed 10,000-observation XAI sample, their Full-condition means are not expected to equal the full-test means in Table 6.

## Appendix H. Post Hoc Search-Output Diagnostics and Equal-Budget Sensitivity Analysis

**Table A10:** Post hoc search-output diagnostics and equal-budget sensitivity analysis.

**(a) Final-Subset Variation Diagnostics**						
**Dataset**	**Unique-Subset Ratio**			**Mean Pairwise Symmetric Difference**		
IoTID20	1.00/1.00			14.44/11.74		
WUSTL-EHMS	0.94/0.72			5.25/4.02		
Edge-IIoTset (ML)	0.88/0.30			3.85/1.48		
RT-IoT2022	1.00/0.98			16.01/13.34		
IoMT-TrafficData	0.88/0.20			3.93/1.36		
Farm-Flow	1.00/0.86			6.08/4.62		
X-IIoTID	1.00/1.00			11.47/8.33		
CICIoV2024	1.00/0.88			7.01/5.71		
**(b) Equal-Budget Sensitivity Check**						
**Dataset**	**Pairs**	**Nogueira (O/HT), All**	**Nogueira (O/HT), Equal**	**ΔF1 Macro**	**ΔMCC**	**ΔRecall Macro**
IoTID20	50	0.051/0.226	0.051/0.226	+0.0002/+0.0002	+0.0003/+0.0003	+0.0003/+0.0003
WUSTL-EHMS	49	0.140/0.332	0.142/0.339	−0.0027/−0.0016	−0.0052/−0.0033	−0.0036/−0.0023
Edge-IIoTset (ML)	20	0.210/0.624	0.196/0.647	+0.0008/+0.0015	+0.0016/+0.0030	+0.0030/+0.0047
RT-IoT2022	50	0.025/0.149	0.025/0.149	+0.0001/+0.0001	+0.0002/+0.0002	+0.0001/+0.0001
IoMT-TrafficData	33	0.131/0.665	0.142/0.669	+0.0001/+0.0001	+0.0002/+0.0002	+0.0001/+0.0001
Farm-Flow	50	0.112/0.349	0.112/0.349	+0.0000/+0.0000	+0.0000/+0.0000	+0.0000/+0.0000
X-IIoTID	49	0.080/0.359	0.078/0.354	+0.0002/+0.0002	+0.0004/+0.0005	+0.0002/+0.0002
CICIoV2024	50	0.119/0.292	0.119/0.292	−0.0019/−0.0019	−0.0037/−0.0037	−0.0038/−0.0038

O: Original AHGA-SA; HT: HTPI-AHGA-SA. Values in Panel (a) and the Nogueira columns are Original AHGA-SA/HTPI-AHGA-SA. For the three Δ columns, values are full-sample/equal-budget estimates, where Δ denotes HTPI-AHGA-SA minus Original AHGA-SA. Unique-subset ratio is the number of distinct selected subsets divided by the 50 runs. Mean pairwise symmetric difference is the mean number of features belonging to only one of two selected subsets across all run pairs. Both are descriptive measures of final-subset variation across runs and do not measure population diversity within a run. Equal-budget pairs are seed pairs for which both configurations used the same number of candidate evaluations. For datasets retaining all 50 pairs, the equal-budget estimates are identical to the full-sample estimates by definition. The equal-budget analysis is a post hoc descriptive sensitivity check and is not interpreted as a causal effect of evaluation budget.
